# The Apical Domain Is Required and Sufficient for the First Lineage Segregation in the Mouse Embryo

**DOI:** 10.1016/j.devcel.2017.01.006

**Published:** 2017-02-06

**Authors:** Ekaterina Korotkevich, Ritsuya Niwayama, Aurélien Courtois, Stefanie Friese, Nicolas Berger, Frank Buchholz, Takashi Hiiragi

**Affiliations:** 1 Developmental Biology Unit, European Molecular Biology Laboratory (EMBL), 69117 Heidelberg, Germany; 2 Medical Systems Biology, UCC, University Hospital and Medical Faculty Carl Gustav Carus, TU Dresden, 01062 Dresden, Germany; 3 Max Planck Institute of Molecular Cell Biology and Genetics, 01307 Dresden, Germany

**Keywords:** early mammalian development, symmetry breaking, reduced systems, cell-fate specification, apico-basal polarity, self-organization

## Abstract

Mammalian development begins with segregation of the extra-embryonic trophectoderm from the embryonic lineage in the blastocyst. While cell polarity and adhesion play key roles, the decisive cue driving this lineage segregation remains elusive. Here, to study symmetry breaking, we use a reduced system in which isolated blastomeres recapitulate the first lineage segregation. We find that in the 8-cell stage embryo, the apical domain recruits a spindle pole to ensure its differential distribution upon division. Daughter cells that inherit the apical domain adopt trophectoderm fate. However, the fate of apolar daughter cells depends on whether their position within the embryo facilitates apical domain formation by Cdh1-independent cell contact. Finally, we develop methods for transplanting apical domains and show that acquisition of this domain is not only required but also sufficient for the first lineage segregation. Thus, we provide mechanistic understanding that reconciles previous models for symmetry breaking in mouse development.

## Highlights

• A reduced system was established to study symmetry breaking in mouse development
• 8-cell stage blastomeres acquire the capacity to self-organize the apical domain
• The apical domain is required and sufficient for the first lineage segregation
• Contact asymmetry specifies cell fate, leading to self-organized embryo patterning

## Graphical Abstract

**Figure:**
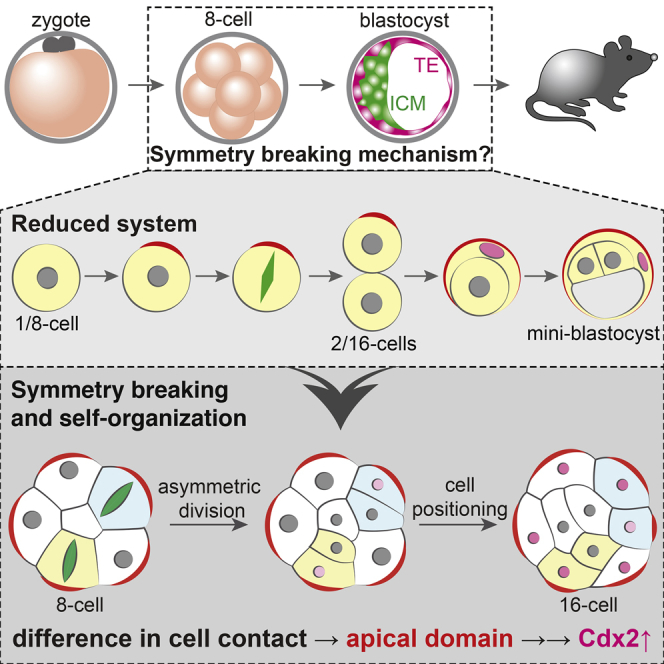

## Teaser

Korotkevich et al. use a reduced system to study symmetry breaking in early mouse development and show that Cdh1-independent contact asymmetry directs temporally controlled apical domain formation, which is necessary and sufficient for segregating the first cell lineages.

## Introduction

Unlike many other model organisms, mammalian eggs lack polarity (Hiiragi and Solter, 2004, Motosugi et al., 2006, Rossant and Tam, 2004). Therefore, blastomeres of the early mammalian embryo are initially equivalent in developmental potency and break their symmetry during pre-implantation development (Rossant and Tam, 2009, Wennekamp et al., 2013, Yamanaka et al., 2006). This symmetry-breaking event results in the bifurcation of the first cell lineages, the inner cell mass (ICM) and the outer extra-embryonic trophectoderm (TE), in the blastocyst. Subcellular localization of Yap is known to distinguish TE and ICM fates (Nishioka et al., 2009): nuclear Yap in outside cells upregulates the expression of Cdx2, a transcription factor essential for TE-fate maturation (Ralston and Rossant, 2008, Strumpf et al., 2005), whereas in inside cells Yap is phosphorylated by Lats and remains cytoplasmic. However, while both cell adhesion (Nishioka et al., 2009) and polarity (Hirate et al., 2013) have been proposed to control the differential localization of Yap, the decisive cue determining Yap localization remains elusive.

The apico-basal cell polarity observed in the 8-cell stage blastomeres and the orientation of their subsequent division have been proposed to play key roles in segregating ICM and TE fates (Johnson and Ziomek, 1981, Johnson, 2009). Blastomeres lacking zygotic copies of aPKC isoforms (*Prkci*^*−*/*−*^;*Prkcz*^*−*/*−*^), which encode apical proteins, show no nuclear Yap, suggesting a defect in TE-fate specification. Nevertheless, the direct relationship between apical polarization and lineage specification remains unclear (Hirate et al., 2013). Alternatively, the position of cells within the embryo, thus the cellular environment rather than the segregation of intracellular determinants, has been proposed to control the ICM- or TE-fate specification (Rossant and Tam, 2009, Tarkowski and Wróblewska, 1967). E-Cadherin (Cdh1)-mediated cell-cell adhesion has indeed been shown to be essential for the correct cell-fate allocation in the blastocyst (Stephenson et al., 2010). However, epithelial polarity is disrupted in blastomeres of maternal and zygotic Cdh1 knockout (*mzCdh1*^*−*/*−*^) embryos, precluding discrimination between the effects of cell-cell adhesion and polarity. Together, the interplay between these two parameters (Cockburn et al., 2013, Hirate et al., 2013), combined with non-stereotypic lineage tree (Dietrich et al., 2015, Morris et al., 2010, Strnad et al., 2016, Watanabe et al., 2014) and stochastic cell-to-cell variability in gene expression (Dietrich and Hiiragi, 2007, Plusa et al., 2008, Ralston and Rossant, 2008), has thus far hindered the identification of the symmetry-breaking cue that segregates the first lineages in mouse development (Wennekamp et al., 2013).

In this study we generate “mini-blastocysts” from single blastomeres isolated from the 8-cell stage embryo (1/8-cell). These develop into doublets and subsequently quadruplets representative of the 16-cell (2/16) and 32-cell (4/32) stages, respectively (Johnson and Ziomek, 1981, Johnson and Ziomek, 1983, Tarkowski and Wróblewska, 1967, Ziomek and Johnson, 1980). Importantly, this “reduced” system recapitulates the lineage segregation of ICM and TE under spatially simplified settings (Anani et al., 2014, Dietrich and Hiiragi, 2007, Maître et al., 2016), thus allowing the decoupling of many interdependent parameters and the individual interrogation of the role of potential cellular cues in this lineage decision. Upon asymmetric division of 1/8-cells, the polar daughter carrying the apical domain begins enveloping its apolar sister. Notably, the former invariably expresses TE-specific transcription factor Cdx2 (Dietrich and Hiiragi, 2007) at a higher level compared with the latter, indicating that cell-fate segregation begins at the 2/16-cell stage, in apparent contrast to the variable Cdx2 expression pattern observed in the 16-cell stage whole embryo (Dietrich and Hiiragi, 2007, Ralston and Rossant, 2008). Thus, we focus on the 1/8-cell to 2/16-cell stage transition to identify the symmetry-breaking cue in the early mouse development and gain mechanistic understanding of patterning in the blastocyst.

## Results

### Acquisition of the Apical Domain Predicts the First Lineage Segregation

**Figure 1:**
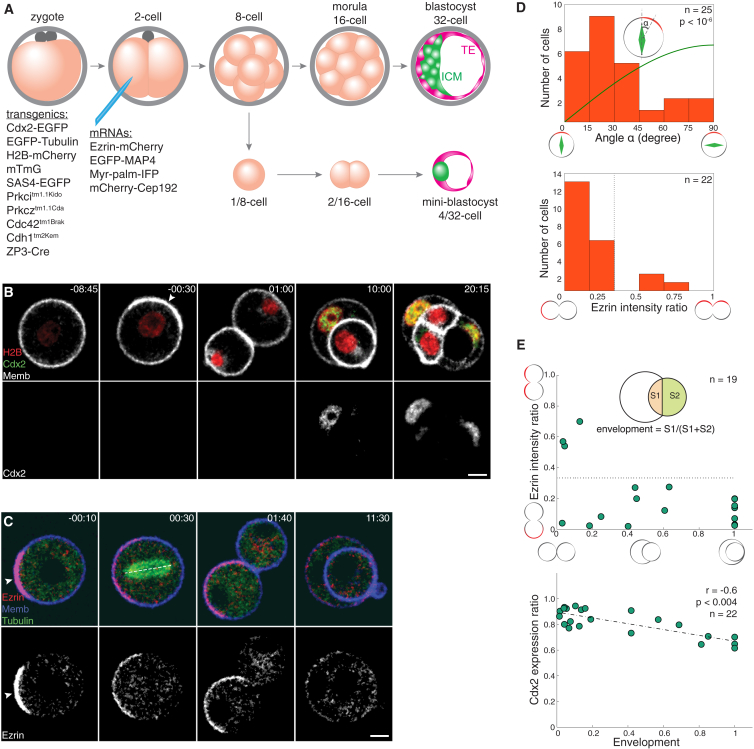
Acquisition of the Apical Domain Predicts the First Lineage Segregation (A) Experimental design. Blastomeres (1/8-cell) are isolated from the 8-cell stage embryo into which mRNAs encoding fluorescent reporters are microinjected at the 2-cell stage. Development of “mini-blastocyst” recapitulates the TE versus ICM lineage segregation. (B) Time-lapse images of the developing 1/8-cell derived from Cdx2-EGFP × R26-H2B-mCherry embryo microinjected with Myr-palm-IFP (Memb) mRNA. The Cdx2 and Memb signals are adjusted differently in the last frame (20:15). (C) Time lapse of 1/8-cell isolated from R26-EGFP-Tuba embryo microinjected with Ezrin-mCherry and Myr-palm-IFP mRNAs. Dashed line denotes spindle. (D) Predominantly asymmetric 1/8-to-2/16-cell divisions as observed by spindle orientation relative to the apical domain (top; green line, random distribution; Kolmogorov-Smirnov test), and as defined by differential distribution of the apical domain (bottom). (E) Upon asymmetric division, polar cells envelop their apolar sister cells (top), the degree of which correlates with the relative level of Cdx2 expression (bottom; Spearman correlation). Arrowheads indicate the apical domain. Time, post-nuclear envelope breakdown (NEBD; hr:min). Scale bars, 10 μm. See also Figures S1 and S2; Movies S1 and S2.

As a basis for investigating the symmetry breaking, we first aimed to identify the earliest cellular parameter that predicts TE-fate specification. Using live imaging, we monitored the development of 1/8-cells derived from embryos where key cellular features were fluorescently labeled (Figure 1A). Blastomeres isolated from the early 8-cell stage embryo initially lacked apico-basal polarity, but subsequent apical domain formation occurred cell-autonomously, as marked with enriched membrane fluorescence intensity (Figures 1B and S1; Movie S1) or Ezrin (Dard et al., 2001, Dard et al., 2009) (Figure S2A; 57%, n = 49 of 86 cells examined), under the temporal control of a developmental program independent of cell cycle or division (Figures S2B and S2C; Kidder and McLachlin, 1985, Levy et al., 1986, Smith and Johnson, 1985). Enrichment of membrane fluorescence in the apical domain is consistent with the presence of microvilli (Ducibella et al., 1977). Unexpectedly, the majority of the blastomeres aligned the mitotic spindle to the apico-basal axis (Figures 1C and 1D; 80% within 0°–45°, n = 20 of 25 cells) and underwent asymmetric division where the apical domain was differentially distributed between the daughter cells (86%, n = 19 of 22 cells with the ratio of Ezrin segregation higher than 3:1, marked with a dotted line in Figures 1D and 1E). After most asymmetric divisions, the cell inheriting the apical domain began enveloping its sister cell (Figures 1C and 1E; n = 14 of 16 cells). The degree of envelopment negatively correlated with the expression level of Cdx2 in polar cells (McDole and Zheng, 2012) (Figures 1B and 1E; n = 22, r = −0.6, p < 0.004). Furthermore, all (n = 16 cells) daughter cells that inherited the apical domain gave rise to cells differentiating to TE. Together these data show that the acquisition of the apical domain predicts the cell division pattern in 1/8-cells, as well as envelopment and TE fate in subsequent stages.

### Apical Domain Is Required for Lineage Segregation and Spindle Orientation

**Figure 2:**
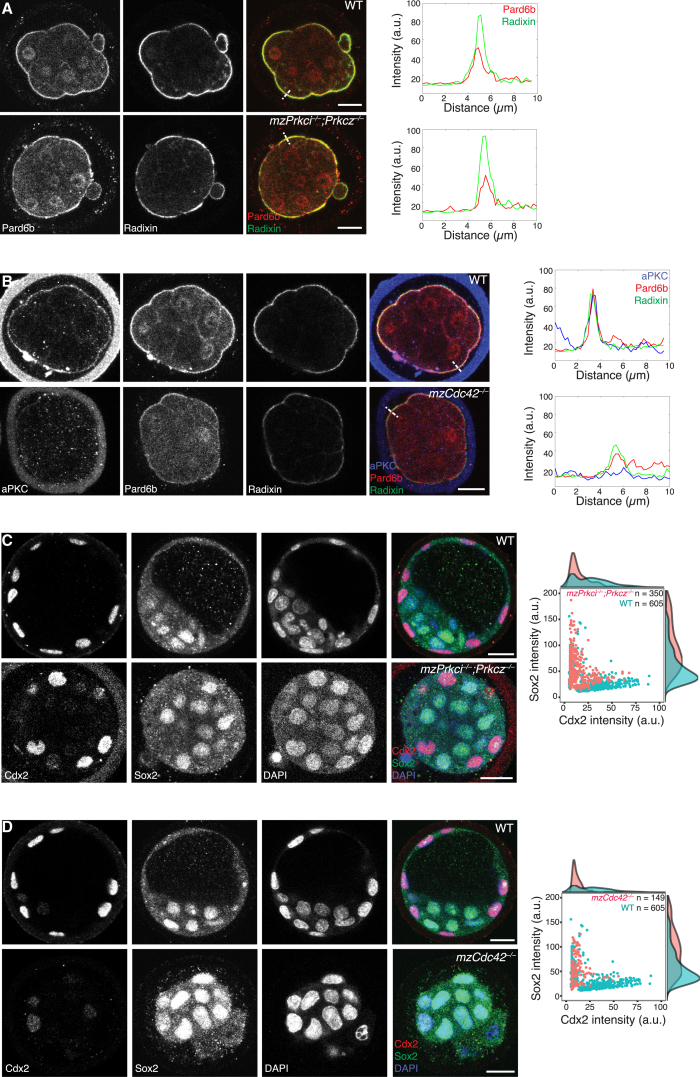
Apical Domain Is Required for Lineage Segregation (A) A single-section immunofluorescence image of WT and *mzPrkci*^*−*/*−*^;*Prkcz*^*−*/*−*^ E3.0 embryos simultaneously stained for Pard6b and Radixin. Intensity profile of Pard6b and Radixin is shown along the dashed lines. (B) A single-section immunofluorescence image of WT and *mzCdc42*^*−*/*−*^ E3.0 embryos simultaneously stained for aPKC, Pard6b, and Radixin. Intensity profile of aPKC, Pard6b, and Radixin is shown along the dashed lines. (C) A single-section immunofluorescence image of WT and *mzPrkci*^*−*/*−*^;*Prkcz*^*−*/*−*^ E4.0 embryos simultaneously stained for Cdx2, Sox2, and DNA (DAPI). Scatter and density plots show fluorescence intensity of Cdx2 and Sox2 for individual blastomeres in WT (n = 605 cells pooled from 11 embryos) and *mzPrkci*^*−*/*−*^;*Prkcz*^*−*/*−*^ (n = 350 cells pooled from 8 embryos) embryos; for Sox2 intensity p < 10^−39^, Mann-Whitney U test. (D) A single-section immunofluorescence image of WT and *mzCdc42*^*−*/*−*^ E4.0 embryos simultaneously stained for Cdx2, Sox2, and DNA (DAPI). Scatter and density plots show fluorescence intensity of Cdx2 and Sox2 for individual blastomeres in WT (n = 605 cells pooled from 11 embryos) and *mzCdc42*^*−*/*−*^ (n = 149 cells pooled from 7 embryos) embryos; for Sox2 intensity p < 10^−16^, Mann-Whitney U test. Scale bars, 20 μm.

These findings encouraged us to investigate whether the apical domain is functionally required and sufficient to induce the lineage segregation process. Requirement of the apical domain for TE-fate specification has so far been tested by examining the subcellular localization of Yap in *zygotic Prkci*^*−*/*−*^;*Prkcz*^*−*/*−*^ embryos (Hirate et al., 2013). When we examined the differential expression of the TE- and ICM-specific transcription factors, however, *mzPrkci*^*−*/*−*^;*Prkcz*^*−*/*−*^ embryos exhibited only mild changes, and indeed apical proteins Pard6b and Radixin remained in the center of the contact-free surface of outer cells (Figures 2A and 2C). To unequivocally examine the consequence of the loss of apical domain, we generated *mzCdc42*^*−*/*−*^ embryos (Wu et al., 2006). In *mzCdc42*^*−*/*−*^ embryos the apical domain is disrupted, as judged by the reduction in the Pard6b, Radixin, and aPKC signals (Figure 2B). These data indicate that Cdc42 controls Pard6b, which then acts upstream of aPKC during de novo apical domain formation in the early mouse embryo. This is in line with the phenotype observed when Pard6b was knocked down by short hairpin RNAs (Alarcón, 2010). In *mzCdc42*^*−*/*−*^ embryos the expression of Cdx2 is diminished and, notably, the majority of blastomeres express Sox2, a marker of the ICM lineage (Figure 2D). These data are consistent with the requirement for the apical domain in TE and ICM lineage segregation.

**Figure 3:**
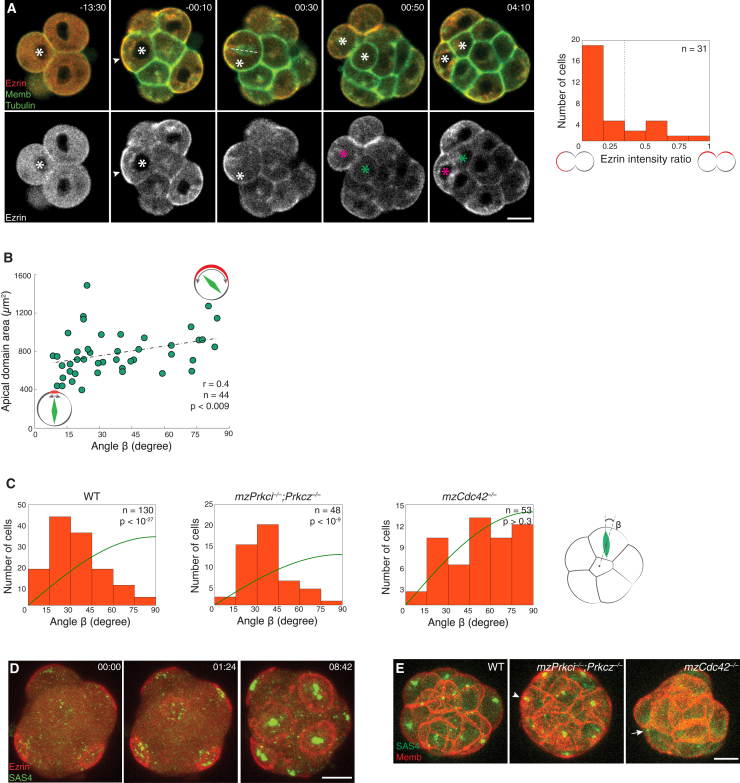
Apical Domain Controls Spindle Orientation (A) Time-lapse images of an asymmetric 8-to-16-cell division (white asterisk) of R26-EGFP-Tuba × mG embryo microinjected with Ezrin-mCherry mRNA, generating a polar (magenta asterisk) and apolar cell (green asterisk). Dashed line denotes spindle. Time, post-NEBD (hr:min). Right: quantification of apical domain distribution during 8-to-16-cell divisions. (B) Scatter plot showing the apical domain surface area and the angle between the spindle and the radial axis of the embryo for blastomeres undergoing 8-to-16-cell divisions in R26-EGFP-Tuba embryos microinjected with Ezrin-mCherry mRNA. Spearman correlation. (C) Spindle orientation of the 8-to-16-cell divisions relative to the radial axis of the embryo in WT, *mzPrkci*^*−*/*−*^;*Prkcz*^*−*/*−*^, and *mzCdc42*^*−*/*−*^ embryos (n = 130, 48 and 53 cells pooled from 17, 6, and 7 embryos, respectively). Green line, random distribution. Kolmogorov-Smirnov test. (D) Maximal-intensity projection (MIP) time-lapse images of the 8-cell stage SAS4-EGFP transgenic embryo microinjected with Ezrin-mCherry mRNA. Time: 00:00 is 68 hr post-hCG (hr:min). (E) MIP live images of the 16-cell stage SAS4-EGFP × mT, *mzPrkci*^*−*/*−*^;*Prkcz*^*−*/*−*^ × SAS4-EGFP × mT and *mzCdc42*^*−*/*−*^ × SAS4-EGFP × mT embryos. Arrow points to off-centered MTOC cluster. Arrowheads indicate the apical domain. Scale bars, 20 μm. See also Figures S3 and S4; Movies S3 and S4.

We next asked whether the apical domain is also required for controlling the spindle orientation during 8-to-16-cell divisions. First, the strong bias toward asymmetric division was confirmed in wild-type (WT) 8-cell stage embryos: 75% (n = 98 of 130 cells in 17 embryos) of blastomeres oriented their spindle along the radial axis of the embryo, and 74% (n = 23 of 31 cells in 5 embryos) underwent asymmetric division (Figures 3A and 3C), in agreement with recent studies (Anani et al., 2014, Watanabe et al., 2014). Furthermore, we found among embryos with naturally varying apical domain size that spindle alignment to the radial axis is more precise the smaller the domain, suggesting that the apical domain controls spindle orientation (Figure 3B; n = 44 cells in 9 embryos, r = 0.4, p < 0.009). Indeed, while *mzPrkci*^*−*/*−*^;*Prkcz*^*−*/*−*^ embryos preferentially aligned their spindle with the radial axis of the embryo, the spindle orientation in *mzCdc42*^*−*/*−*^ embryos was randomized, further supporting the notion that the apical domain is required for spindle orientation (Figure 3C). This role of the apical domain may be shared with other epithelial cells through centrosome recruitment to control spindle orientation (Hebert et al., 2012, Lechler and Fuchs, 2005, Schober et al., 1999). To understand the mechanism by which the apical domain controls spindle orientation in mouse pre-implantation embryos that lack centrioles, we examined the dynamics of microtubule organizing centers (MTOCs) that play a key role in acentrosomal spindle assembly (Courtois et al., 2012). Live imaging of the SAS4-EGFP embryo (Figure S3) revealed that when the apical domain emerges de novo at the 8-cell stage in the center of the contact-free surface, MTOCs cluster to the subapical region and eventually form one spindle pole (Courtois et al., 2012, Houliston et al., 1987) (Figures S4 and 3D; Movie S3). In *mzCdc42*^*−*/*−*^ but not *mzPrkci*^*−*/*−*^;*Prkcz*^*−*/*−*^ embryos, formation of the subapical MTOC cluster was diminished (Figure 3E and Movie S4), indicating that MTOCs are recruited by the emerging apical domain. Collectively, the apical domain ensures its differential distribution between daughter cells during 8-to-16-cell divisions and plays an essential role for TE and ICM lineage segregation.

### Apical Domain Is Sufficient for Initiating Cell-Fate Segregation

**Figure 4:**
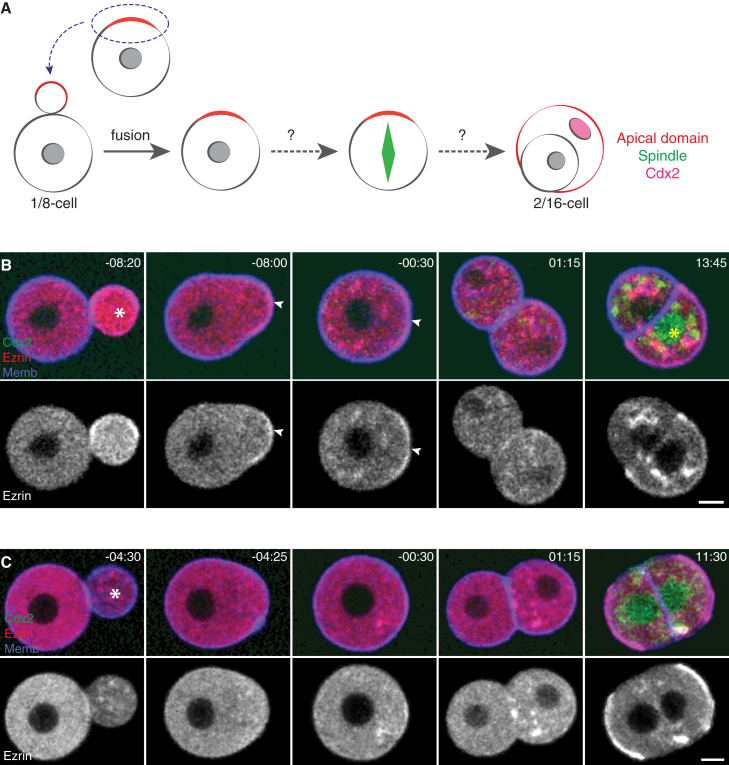
Apical Domain Is Sufficient for Initiating Cell-Fate Segregation (A) Experimental design. The apical domain is transplanted into an apolar 8-cell stage blastomere to test whether it can induce the cell lineage segregation process. (B) Time lapse of an 8-cell stage blastomere isolated from Cdx2-EGFP embryo microinjected with Ezrin-mCherry and Myr-palm-IFP mRNAs, developing after integration of a cell fragment (white asterisk) derived from an 8-cell stage blastomere containing the apical domain (note Cdx2 expression marked by yellow asterisk). Arrowheads indicate the apical domain. (C) Time lapse of an 8-cell stage blastomere isolated from Cdx2-EGFP embryo microinjected with Ezrin-mCherry and Myr-palm-IFP mRNAs, developing after integration of a cell fragment (asterisk) derived from an 8-cell stage blastomere containing the non-apical domain. Time, post-NEBD (hr:min). Scale bars, 10 μm. See also Movie S5.

Next, to examine sufficiency of the apical domain for TE-fate specification, we developed methods that allow us to transplant the apical domain of a polarized 8-cell stage blastomere into a yet apolar 8-cell stage blastomere (Figure 4A). Transplanted apical domains were integrated into apolar blastomeres and stably maintained, as monitored by the Ezrin signal (Figure 4B and Movie S5; n = 12 of 14 cells). By contrast, integration of a cell fragment derived from the non-apical domain did not induce apical domain formation at the fusion site (Figure 4C; n = 4 of 4 cells). Remarkably, the transplanted apical domain induced asymmetric division (n = 12 of 12 cells), and the daughter cell inheriting the apical domain became committed to the TE lineage, as judged by cell envelopment process or differential Cdx2 expression (Figure 4B and Movie S5; n = 9 of 12 cells). Combined, these data provide the first experimental evidence that acquisition of the apical domain is not only required but also sufficient for initiating cell-fate segregation.

**Figure 5:**
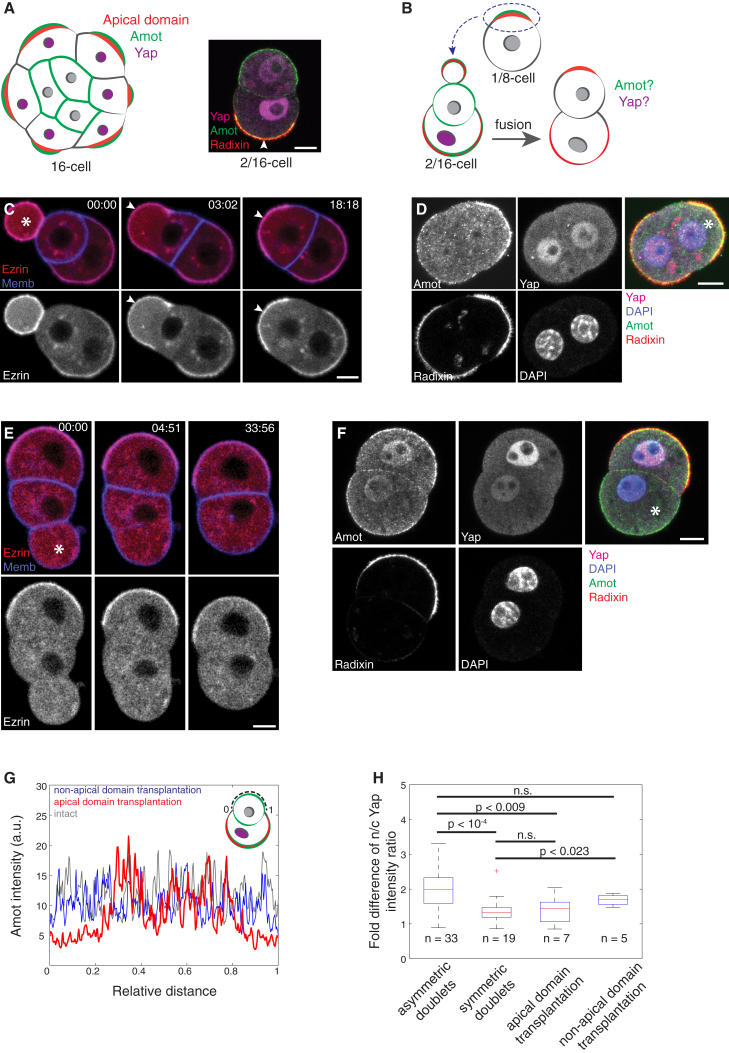
Apical Domain Controls Cell Fate through Yap Signaling (A) Schematic and immunofluorescence staining for subcellular distribution of Amot, Yap, and Radixin (apical domain) in the 16-cell stage embryo and in a 2/16-doublet, respectively. (B) Experimental design. The apical domain is transplanted into an apolar cell of 2/16-doublet to test whether it can change subcellular distribution of Amot and Yap. (C) Time lapse of a 2/16-cell doublet isolated from the embryo microinjected with Ezrin-mCherry and Myr-palm-IFP mRNAs, developing after integration of a cell fragment (white asterisk) derived from an 8-cell stage blastomere containing the apical domain. (D) Immunofluorescence image of the 2/16-cell doublet after transplantation of apical domain (recipient cell marked by asterisk) stained for Amot, Yap, Radixin, and DNA (DAPI). (E) Time lapse of a 2/16-cell doublet isolated from the embryo microinjected with Ezrin-mCherry and Myr-palm-IFP mRNAs, developing after integration of a cell fragment (asterisk) derived from an 8-cell stage blastomere containing the non-apical domain. (F) Immunofluorescence image of the 2/16-cell doublet after transplantation of the non-apical domain (recipient cell marked by asterisk) stained for Amot, Yap, Radixin, and DNA (DAPI). (G) Cortical intensity profiles under the dashed line of apolar recipient cell shown in (A), (D), and (F). (H) Box plot showing the nucleus-to-cytoplasm (n/c) Yap intensity ratio of polar cell divided by that of its sister cell in the respective 2/16-cell doublet. Mann-Whitney U test. In the box plot, the central mark indicates the median, with the bottom and top edges of the box indicating the 25^th^ and 75^th^ percentiles, respectively. The whiskers extend to the most extreme data points, with exception of the outliers that are marked individually with the + symbol. n.s., not significant. Arrowheads indicate the apical domain. Time, post-fusion (min:s). Scale bars, 10 μm.

We then addressed the mechanism by which inheritance of the apical domain influences cell-fate specification. Polarity-dependent localization of Angiomotin (Amot) was shown to control subcellular localization of Yap (Hirate et al., 2013). To test whether the ectopic apical domain induces a change in the subcellular localization of Amot and Yap, we designed another apical transplantation experiment using asymmetric 2/16-cell doublets. In polar cells of 2/16-doublets Amot is localized to the apical domain and Yap is predominantly nuclear, in agreement with findings in the whole embryo (Hirate et al., 2013). In contrast, their apolar partners localize Amot to the entire membrane, while Yap is mostly cytoplasmic (Figure 5A; n = 33 doublets). These data suggest that cell polarity but not adhesion controls differential Amot and Yap localization. When unperturbed, the apolar cell of a 2/16-cell doublet would eventually be enveloped by its polar sister and give rise to ICM. We transplanted the apical domain into apolar cells of 2/16-doublets and asked whether it induces changes in Amot and Yap distribution and subsequent cell fate (Figure 5B). Upon transplantation of the apical (Figure 5C; n = 7 of 7 cells) but not non-apical domain (Figure 5E; n = 5 of 5 cells), we observed relocation of Amot to the ectopic apical region as well as nuclear translocation of Yap (Figures 5D and 5F–5H), suggesting that the apical domain controls cell fate through Yap signaling. Notably, this change in subcellular localization of Amot and Yap does not require cell division (Figures 5C and 5D), indicating that it is the acquisition of the apical domain rather than its asymmetric distribution that induces the TE-fate differentiation.

### Cell Position and Cdh1-Independent Cell Contact Direct Apical Domain Formation and TE-Fate Specification

**Figure 6:**
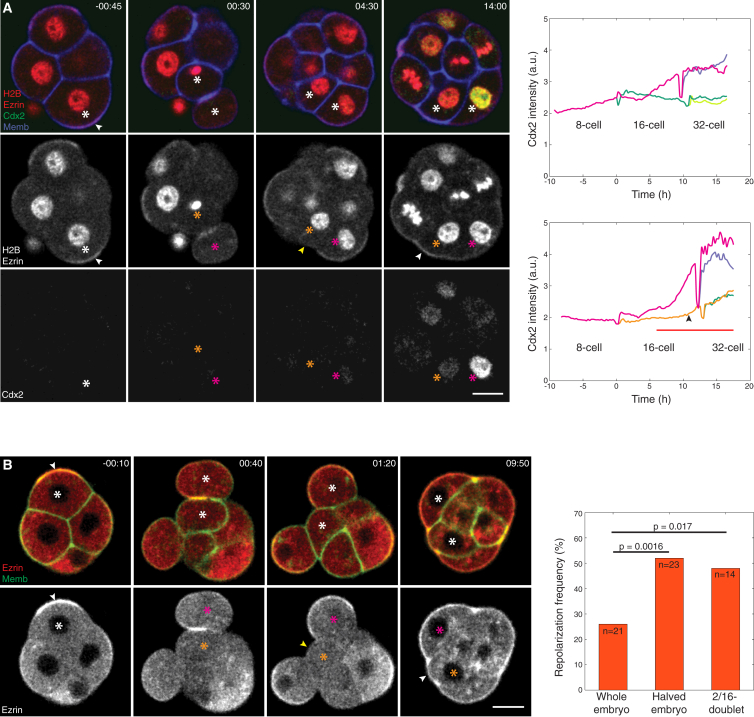
Cell Position Regulates Apical Domain Formation and Fate Specification (A) Time lapse of asymmetric 8-to-16-cell division (white asterisk; generating cells marked with orange and magenta asterisks) of Cdx2-EGFP × R26-H2B-mCherry embryo microinjected with Ezrin-mCherry and Myr-palm-IFP mRNAs, in which an apolar cell (orange asterisk) acquires the apical domain (from yellow to white arrowheads) and begins expressing Cdx2. Dynamics of Cdx2 expression in (top) an 8-cell stage blastomere undergoing asymmetric division generating one TE- and the other ICM-forming cell, and (bottom) another 8-cell stage example, as shown in the top panels, with line colors indicating the cells marked by asterisks of the same color. Cdx2 expression is upregulated (black arrowhead) after an apolar cell acquires the apical domain (red underline). (B) Time lapse of the halved embryo in which an apolar cell generated after 4/8-to-8/16-cell division (white asterisk; generating cells marked with orange and magenta asterisks) acquires the apical domain (from yellow to white arrowheads). The Memb signals are adjusted differently in the last frame (09:50). The ratio of initially apolar cells that acquire the apical domain is different between the whole, halved, and 2/16-embryos. Two-tailed Fisher's exact test. Magenta asterisk, polar cell. Time, post-NEBD (hr:min). Scale bars, 20 μm. See also Figure S5.

High-resolution live imaging in whole embryos showed that upon asymmetric divisions, a few apolar daughter cells remained at or were repositioned to the embryo surface, and acquired an apical domain (Figure S5A; 23%, n = 21 of 92 apolar cells in 16 embryos). The majority of these cells eventually turned on Cdx2 expression, although later than cells directly inheriting the apical domain upon the 8-to-16-cell division (Figure 6A; n = 11 of 13 cells in 8 embryos). Combined, these data consistently indicate that acquisition of the apical domain induces the TE differentiation and that division orientation alone cannot determine cell fate, in agreement with recent studies (McDole et al., 2011, Watanabe et al., 2014, Yamanaka et al., 2010). The rate of repolarization was significantly higher in halved (8/16-cell) and 2/16-cell embryos (Figures 6B and S5B; 51%, n = 23 of 45 apolar cells in 13 embryos and 48%, n = 14 of 29 apolar cells in 29 asymmetric 2/16-doublets), suggesting that cell positioning is indeed not pre-determined.

**Figure 7:**
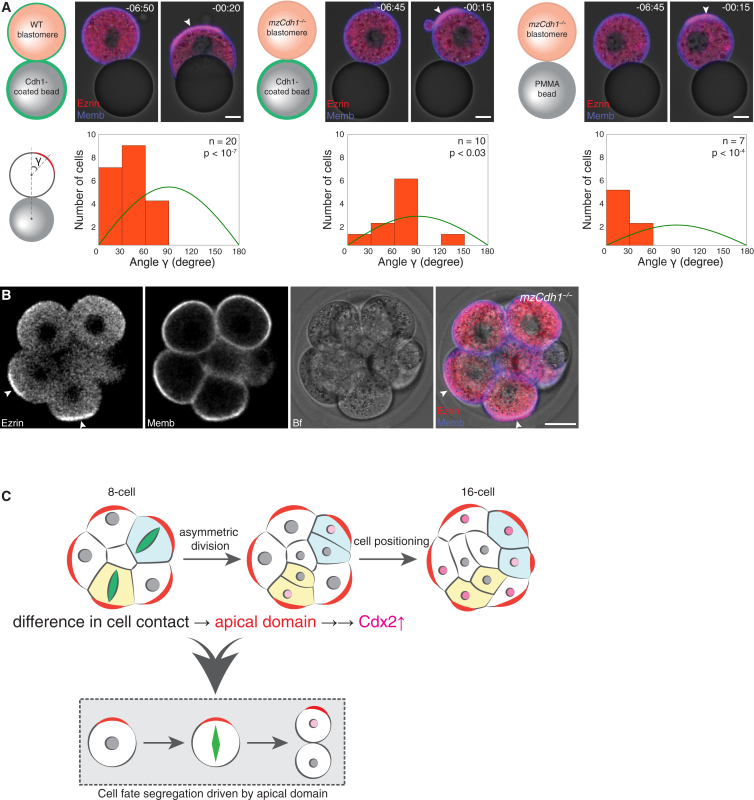
Cdh1-Independent Cell Contact Directs Apical Domain Formation (A) Cdh1-coated beads induce the apical domain opposite to the contact in WT 1/8-cell, as well as *mzCdh1*^*−*/*−*^ cell, microinjected with Ezrin-mCherry and Myr-palm-IFP mRNAs. PMMA beads also induce the apical domain in *mzCdh1*^*−*/*−*^ cells. Time, post-NEBD (hr:min). Scale bars, 10 μm. Lower panels: quantification of apical domain position relative to the contact with a bead. Green line, random distribution. Kolmogorov-Smirnov test. (B) Live image of the 8-cell stage *mzCdh1*^*−*/*−*^ embryo microinjected with Ezrin-mCherry and Myr-palm-IFP mRNAs. Scale bar, 20 μm. (C) Model of symmetry breaking in mouse development. The presence of contact-free cell surface in outside cells directs formation of the apical domain that, in turn, induces asymmetric division and TE-fate specification. Arrowheads indicate the apical domain. See also Figure S6 and Movie S6.

Given the importance of cell position and the apical domain in cell-fate specification, we wished to examine the mechanistic link between these two factors. Cell-cell contact has been shown to induce the formation of the apical domain opposite to the contact point (Ziomek and Johnson, 1980). In particular, Cdh1 was proposed to play a key role in orienting the axis of cell polarization (Johnson et al., 1986) and in fate allocation (Stephenson et al., 2010). Here again we took advantage of the reduced experimental system and used cell-sized beads coated with specific molecules to mimic cell contact in a molecularly defined manner. Using early apolar 1/8-cells, we first observed that a bead coated with Cdh1 potentiates apical domain formation opposite the point of contact more efficiently than when cells are left in isolation (Figure 7A; 84%, n = 47 of 56 cells, compared with 57% as shown in Figure S2A) (Ziomek and Johnson, 1980). To examine whether this contact specifically requires Cdh1, we repeated the experiment with 1/8-cells derived from *mzCdh1*^*−*/*−*^ embryos (Stephenson et al., 2010). Unexpectedly, *mzCdh1*^*−*/*−*^ 1/8-cells adhered to the Cdh1-coated beads and formed their apical domain opposite the contact point (Figure 7A; 85%, n = 11 of 13 cells), suggesting that Cdh1 is dispensable for directing the apical domain formation. This was further supported by using uncoated beads made of polymethyl methacrylate (PMMA) (Figure 7A; 88%, n = 7 of 8 cells) to which *mzCdh1*^*−*/*−*^ cells also adhered. Furthermore, *mzCdh1*^*−*/*−*^ embryos are capable of assembling the apical domains away from cell-cell contacts during the 8-cell stage (Figure 7B and Movie S6; n = 5 embryos), in agreement with an earlier study (Stephenson et al., 2010). These findings consistently indicate that cell contact, independent of Cdh1, breaks cellular symmetry and directs apico-basal polarization. Apical domain formation is thus induced in WT 1/8-cells upon contact with uncoated PMMA beads, preferentially on the opposite side of the contact point (Figures S6A and S6B; n = 24 cells). This is in line with our finding that the apical domain emerges at the center of the contact-free surface (Figure S4). Again, this apical domain induces asymmetric division (Figures S6A and S6B; n = 17 of 26 cells) and specific upregulation of Cdx2 in polar daughter cells (Figure S6C; n = 20 cells).

## Discussion

Taken together, our data unveil novel mechanistic insights into symmetry breaking in mouse embryogenesis. Cells in the early mouse embryo acquire the capacity to self-organize the apical domain when they reach the 8-cell stage (Figure S2C). Cell contacts, when available, facilitate and direct apical domain formation, which is in turn both necessary and sufficient for segregating the first cell lineages (Figure 7C). Cell position within the embryo, specifically the absence (inside) or presence (outside) of a contact-free surface, determines whether it acquires an apical domain, thus determining its eventual fate. This model is conceptually reminiscent of the inside-outside model, in which cell differentiation was proposed to be dependent on its position within the embryo (Tarkowski and Wróblewska, 1967). However, the mechanism by which a cell recognizes its position had remained elusive. Our present data collectively suggest that difference in cell-surface contact is the crucial signal distinguishing “outside” from “inside” positions, and we unexpectedly identified that Cdh1 is dispensable in this context. Furthermore, we used the reduced experimental system to demonstrate that lineage segregation is driven by the apical domain. This is not predicted by the inside-outside model but is in line with the cell polarity model (Johnson and Ziomek, 1981), in which cell fate was proposed to be determined by distribution of the apical domain between daughter cells. However, the high number of asymmetric divisions leaves more apolar cells than could be accommodated within the 16-cell stage embryo (Anani et al., 2014, Dietrich and Hiiragi, 2007). While the cell polarity model attributes the higher number of polar cells in the embryo to “symmetric” divisions, we observe that apolar cells compete for inside positions, and those that are pushed out to the surface give rise to polarized TE. We therefore clarify the apparent disparity between the cell polarity model (Johnson and Ziomek, 1981) and current live-imaging studies (Anani et al., 2014, Watanabe et al., 2014). Notably, our data also explain cell-to-cell Cdx2 expression heterogeneity observed in outside cells of the 16-cell stage embryo (Dietrich and Hiiragi, 2007, Dietrich et al., 2015, Ralston and Rossant, 2008). Our model thus reconciles the earlier inside-outside and cell polarity models as well as recent data.

The exact nature of cell contact that can direct the apical domain assembly is the subject of future studies. The cell contact may elicit signaling via cell-surface adhesion molecules. P-cadherin (Cdh3) was shown to be expressed in the mouse pre-implantation embryo (Stephenson et al., 2010) and could compensate for the loss of Cdh1. Alternatively, the cell contact could induce mechanical change, e.g., deformation of cell shape or local change in cortical contractility (Maître et al., 2015), that may influence apical domain assembly. In any case our experimental system will allow for exploring the mechanisms underlying the de novo formation of epithelial polarity and the signals with which cell contacts control this process.

Determination of cell fate in the embryo ultimately depends on cell positioning and spatial context. A cell may read its position within the embryo through contact asymmetry and transduce the signal for cell-fate specification by forming an apical domain on the contact-free surface as shown in this study, or by mechanosensing (Maître et al., 2016). Our recent study also showed that the apical domain exhibits reduced cortical contractility and its asymmetric distribution generates daughter blastomeres of distinct contractilities, leading to cell sorting within the embryo (Maître et al., 2016). These feedback interactions between cell contact, polarity, mechanics, and fate may be key principles underlying multi-cellular self-organization. Further studies based on this model will unveil an integrated picture of symmetry breaking and self-organization in early mammalian development.

## STAR★Methods

### Key Resources Table

**Table:** 

Reagent or Resource	Source	Identifier
**Antibodies**		

Mouse anti-Cdx2	BioGenex	MU392-UC; RRID: AB_2335627
Goat anti-Sox2	Santa Cruz Biotechnology	sc-17320; RRID: AB_2286684
Rabbit anti-aPKC	Santa Cruz Biotechnology	sc-216; RRID: AB_2300359
Mouse anti-aPKC	Santa Cruz Biotechnology	sc-17781; RRID: AB_628148
Rabbit anti-Pard6b	Santa Cruz Biotechnology	sc-67393; RRID: AB_2267889
Rat anti-Radixin	Kikuchi et al., 2002	N/A
Rabbit anti-Amot	Hirate et al., 2013	N/A
Mouse anti-Yap	Abnova	H00010413-M01; RRID: AB_535096
Rat anti-tyrosinated γ-Tubulin	AbD Serotec	MCA77G; RRID: AB_325003
Mouse anti-Pcnt	BD	611814; RRID: AB_399294
Rabbit anti-SAS4	Basto et al., 2006	N/A

**Chemicals, Peptides, and Recombinant Proteins**		

Pregnant mare’s serum gonadotropin	Intervet	Intergonan
Human chorionic gonadotropin	Intervet	Ovogest 1500
KSOMaa	Zenith biotech	ZEKS-050
KSOMaa with HEPES	Zenith biotech	ZEHP-050
KSOMaa without Ca^2+^ and Mg^2+^	Custom-made	N/A
Mineral oil	Sigma	M8410
Silicone oil	Ibidi	50051
Hyaluronidase	Sigma	H4272
Proteinase K	Sigma	P8811
PVP-40	Sigma	P0930
Aphidicolin	Sigma	A0781
Cytochalasin D	Sigma	C8273
Recombinant mouse Cdh1-Fc chimera protein	RnDsystems	748-EC-050
BSA	Sigma	A3311

**Critical Commercial Assays**		

Sendai virus envelope	Cosmo Bio Co.	ISK-CF-001-EX
mMessage mMachine transcription kits	Ambion	AM1340, AM1348, AM1344, AM1345
Poly(A)Tailing Kit	Ambion	AM1350

**Experimental Models: Cell Lines**		

R1/E ES cells	Transgenic Core Facility at Max Planck Institute of Molecular Cell Biology and Genetics	N/A

**Experimental Models: Organisms/Strains**		

Mouse: (C57BL/6xC3H) F1	Laboratory Animal Resources at the European Molecular Biology Laboratory	N/A
Mouse: C57BL/6	Laboratory Animal Resources at the European Molecular Biology Laboratory	N/A
Mouse: CD1	Laboratory Animal Resources at the European Molecular Biology Laboratory	N/A
Mouse: Cdx2-EGFP knock-in	K. McDole; McDole and Zheng, 2012	N/A
Mouse: R26-EGFP-Tuba	Laboratories of Animal Resource development and Genetic Engineering, RIKEN Center for Life Science Technologies; Abe et al., 2011	CDB0245K
Mouse: R26-H2B-mCherry	Laboratories of Animal Resource development and Genetic Engineering, RIKEN Center for Life Science Technologies; Abe et al., 2011	CDB0239K
Mouse: mTmG	The Jackson Laboratory; Muzumdar et al., 2007	007676
Mouse: Cdc42^tm1Brak^	C. Brakebusch; Wu et al., 2006	N/A
Mouse: Prkci^tm1.1Kido^	H. Sasaki; Hirate et al., 2013	N/A
Mouse: Prkcz^tm1.1Cda^	H. Sasaki; Hirate et al., 2013	N/A
Mouse: Cdh1^tm2Kem^	The Jackson Laboratory; Boussadia et al., 2002	005319
Mouse: ZP3-Cre	The Jackson Laboratory; Lewandoski et al., 1997	003394
Mouse: SAS4-EGFP BAC transgenic	This study	N/A

**Recombinant DNA**		

pc31-mCherry-Cep192	M. Schuh	N/A
pGEMHE-EGFP-MAP4	J. Ellenberg	N/A
pGEMHE-Myr-palm-IFP670	J. Ellenberg	N/A
pRN3-Ezrin-mCherry	S. Louvet-Vallée	N/A
RP11-756A22 BAC	BACPAC Resource Center (BPRC)	N/A
LAP tagging cassette	Poser et al., 2008	N/A

**Sequence-Based Reagents**		

Primer: LAP cassette integration PCR, forward: TGCTCTACGGCTGATGTGTC (hSASS4-F)	This paper	N/A
Primer: LAP cassette integration PCR, reverse: TGCAAACGGTCATCAAGAAA (hSASS4-R)	This paper	N/A
See Table S1 for genotyping primer list	N/A	N/A

**Software and Algorithms**		

Matlab	MathWorks	https://www.mathworks.com/products/matlab.html
R		https://www.r-project.org
Fiji	Schindelin et al., 2012	https://fiji.sc
Imaris	BITPLANE	http://www.bitplane.com/imaris/imaris
Ilastik	Sommer et al., 2011	http://ilastik.org
StarryNite	Boyle et al., 2006	http://waterston.gs.washington.edu
AceTree	Boyle et al., 2006	http://waterston.gs.washington.edu
AutofocusScreen	Rabut and Ellenberg, 2004	http://www.ellenberg.embl.de/index.php/software/microscopyautomation

**Other**		

PMMA microbeads	Microparticles	PMMA-R-B375
Protein A-coated PMMA microbeads	Microparticles	PMMA-Protein A-S2976B
Plastic-bottom dish	Ibidi	81151
Tissue culture uncoated dish	Ibidi	81501

### Contact for Reagent and Resource Sharing

Further information and requests for resources and reagents should be directed to and will be fulfilled by the Lead Contact Takashi Hiiragi (hiiragi@embl.de).

### Experimental Model and Subject Details

#### Animal Work

All animal work was performed in the Laboratory Animal Resources (LAR) at the European Molecular Biology Laboratory, with permission from the institutional veterinarian overseeing the operation (ARC number TH11 00 11). The animal facilities are operated according to international animal welfare rules (Federation for Laboratory Animal Science Associations guidelines and recommendations). Mouse colonies are maintained in specific pathogen-free conditions with 12-12 hrs light-dark cycle. All mice used for experiments were at least 7 weeks old.

#### Transgenic Mice and Genotyping

The following mouse lines were used in this study: (C57BL/6xC3H) F1 for WT, Cdx2-EGFP knock-in (McDole and Zheng, 2012), R26-EGFP-Tuba, R26-H2B-mCherry (Abe et al., 2011), mTmG (Muzumdar et al., 2007), Cdc42^tm1Brak^ (Wu et al., 2006), Prkci^tm1.1Kido^, Prkcz^tm1.1Cda^ (Hirate et al., 2013), Cdh1^tm2Kem^ (Boussadia et al., 2002), ZP3-Cre (Lewandoski et al., 1997) and SAS4-EGFP BAC transgenic mice.

To generate SAS4-EGFP mice the SAS4 gene was modified on a bacterial artificial chromosome by recombineering (Testa et al., 2003). The stop codon of the SAS4 coding sequence in the RP11-756A22 BAC was replaced with the LAP cassette (Poser et al., 2008). The LAP tagging cassette consists of EGFP sequence followed by an internal ribosome entry site and the neomycin-kanamycin resistance gene for eukaryotic and bacterial expression (Figure S3A). A correct placement of the tagging cassette was confirmed by PCR amplifying the integration site using TGCTCTACGGCTGATGTGTC (hSASS4-F) and TGCAAACGGTCATCAAGAAA (hSASS4-R) primers producing a 3500 bp fragment. To generate a transgenic ES cell line the modified BAC was transfected into R1/E ES cells that were selected for BAC integration with 250 μg/ml G418 (Invitrogen, 10131-019). The ES cells were subsequently injected into C57BL/6 blastocysts that were transferred into pseudo-pregnant CD1 female mice. The resultant pups were examined for the presence of BAC integration by genotyping.

Mice were genotyped using standard tail genotyping procedures (for genotyping primers and fragment sizes see Table S1).

*mzPrkci*^*−*/*−*^;*Prkcz*^*−*/*−*^ embryos were generated by mating *Prkci*^*floxed*/*floxed*^;*Prkcz*^*−*/*−*^ x Zp3Cre^tg/+^ female with *Prkci*^*+*/*−*^;*Prkcz*^*−*/*−*^ male mice (Lewandoski et al., 1997). For experiments in Figures 2A, 3C, and 3E *mzPrkci*^*−*/*−*^;*Prkcz*^*−*/*−*^ embryos were genotyped by single embryo PCR as described before (Dietrich et al., 2015). Briefly, single embryos were transferred into 10 μl of lysis buffer (PCR buffer (Fermentas, EP0402) supplemented with 0.2 μg/μl proteinase K (Sigma, P8811), incubated at 55°C for 1 h and then at 96°C for 10 min. Two to 10 μl of the lysate was used for PCR. For experiments in Figure 2C embryos were genotyped by immunofluorescence staining for aPKC.

*mzCdc42*^−/−^ embryos were generated by mating *Cdc42*^*floxed*/*floxed*^ x Zp3Cre^tg/+^ female with *Cdc42*^*+*/−^ male mice. For all experiments *mzCdc42*^−/−^ embryos were genotyped by single embryo PCR.

*mzCdh1*^−/−^ embryos were generated by mating *Cdh1*^*floxed*/*floxed*^ x Zp3Cre^tg/+^ female with *Cdh1*^*+*/−^ male mice. For experiments in Figure 7A embryos were recovered at embryonic day 1.5 (E1.5) and one of the two blastomeres was injected with mRNAs encoding Ezrin-mCherry and Myr-palm-IFP. At the late 4-cell stage the zona pellucida was removed mechanically (Tsunoda et al., 1986) and embryos were dissociated into 4x 1/4-cell blastomeres. Two non-injected 1/4-cell blastomeres were reaggregated to form half embryos and cultured further to determine the genotype. The remaining 2x injected 1/4-cell blastomeres were dissociated again at the 2/8-cell stage into 4x 1/8-cells and used for experiments. *mzCdh1*^−/−^ embryos or half embryos were cultured to blastocyst stage and then genotyped by their ability or inability to form the blastocyst and by single embryo PCR.

#### Mouse Embryos

To obtain pre-implantation embryos, female mice were superovulated by intraperitoneal injection of 5 international units (IU) of pregnant mare’s serum gonadotropin (Intervet, Intergonan) followed by 5 IU human chorionic gonadotropin (hCG; Intervet, Ovogest 1500) 48-50 hrs later, and mated with male mice. Zygotes were recovered at E0.5 by ripping the ampula of the oviduct recovered from the pregnant female mice in KSOMaa with HEPES (H-KSOMaa; Zenith biotech, ZEHP-050) supplemented with 300 μg/ml hyaluronidase (Sigma, H4272) and 10 mg/ml PVP-40 (Sigma, P0930). Two- and 8-cell stage embryos were obtained by flushing the oviduct with H-KSOMaa at E1.5 and E2.5, respectively. Morulae and blastocysts were obtained by flushing the uterus with H-KSOMaa at E3.0-E4.0. After recovery embryos were washed in H-KSOMaa, transferred into 10 μl drops of KSOMaa (Zenith biotech, ZEKS-050) covered with mineral oil (Sigma, M8410) on a tissue culture dish (Falcon, 353001), and cultured in a CO_2_ incubator (Thermo Scientific, Heracell 240i) at 37°C with 5% CO_2_.

### Method Details

#### Chemical Reagents

Aphidicolin (Sigma, A0781) 2.5 mg/ml dimethyl sulfoxide (DMSO) stock was diluted to 0.5 μg/ml in KSOMaa. Embryos were placed in medium containing aphidicolin or an equivalent amount of DMSO alone.

Cytochalasin D (CCD; Sigma, C8273) 10 mM DMSO stock is diluted to 10 μM in H-KSOMaa. Isolated blastomeres are placed in medium containing CCD under mineral oil.

#### Microinjection of mRNAs

For in vitro transcription plasmids were linearized using restriction enzymes. mRNA was transcribed in vitro using mMessage mMachine transcription kits (Ambion, AM1340, AM1348, AM1344, AM1345), followed by poly-adenylation using Poly(A)Tailing Kit (Ambion, AM1350).

**Table:** In Vitro mRNA Preparation

Construct	Linearization Enzyme	Kit	Polyadenylation	mRNA Concentration Used for Injections, ng/μl
pc31-mCherry-Cep192	NotI	T7	+	140
pGEMHE-EGFP-MAP4	SphI	T7	-	130
pGEMHE-Myr-palm-IFP670	PacI	T7 ULTRA	+	30
pRN3-Ezrin-mCherry	SfiI	T3	-	150

Microinjection of mRNAs into embryos was performed on epifluorescence microscope (Zeiss, Observer.Z1) equipped with an injector (Eppendorf, FemtoJet) and micromanipulators (Narishige, MON202-D) maintained at 32°C in an incubation chamber. Microinjection needles (Warner Instruments, G100TF-6) and holding pipettes (Warner Instruments, GC100T-15) were prepared using a micropipette puller (Sutter Instrument, P-97) and a microforge (Narishige, MF-900). During microinjection embryos were kept in 10 μl H-KSOMaa drop covered with mineral oil on a glass-bottom dish (MatTek, P506-1.5-14-F). mRNAs were injected into cytoplasm of the zygote or 2-cell stage embryo, one cell or both cells depending on the experimental condition.

#### Micromanipulation

##### Isolation of the Blastomere

To dissociate blastomeres from the embryo, the zona pellucida was first removed mechanically (Tsunoda et al., 1986) or by 3-min incubation with pronase (0.5% w/v Proteinase K in H-KSOMaa supplemented with 0.5% PVP-40). Subsequently the embryos were placed for 10 min into KSOMaa medium without Ca^2+^ and Mg^2+^ (Biggers et al., 2000), and blastomeres were dissociated into single cells by pipetting up and down with a narrow flame-polished glass capillary tube (Brand, 708744).

**Table:** Preparation of KSOMaa Medium without Ca^2+^ and Mg^2+^

Component	Amount to Add for 1L Solution	Source	Identifier
BSA	1 g	Sigma	A3311
NaCl	5.55 g	Sigma	S5886
KCl	0.186 g	Fisher	P217-500
KH_2_PO_4_	0.0476 g	Fisher	P285-500
Na Lactate	1.43 ml of 60% syrup	Sigma	L7900
D(+) glucose	0.036 g	Sigma	G6152
Penisilin and Streptomycin	10 ml	GIBCO	15070-063
NaHCO_3_	2.1 g	Sigma	S5761
Phenol red	0.01 g	Sigma	P5530
Na pyruvate	0.022 g	Sigma	P4562
Na_4_EDTA ⋅ 4H_2_O	0.00452 g	Sigma	E5391
MEM essential AA solution	10 ml	GIBCO	11130-036
Non-essential AA solution	5 ml	GIBCO	11140-035
L-Glutamine solution	5 ml	GIBCO	25030-032

Embryos without the zona pellucida and isolated blastomeres were cultured in Petri dishes (Falcon, 351008) to minimize attachment to the bottom of the dish. For experiments requiring polarized blastomeres, embryos were dissociated at the late 8-cell stage. Non-polarized blastomeres were obtained by first dissociating embryos at the late 4-cell stage. Recovered 1/4-cells were inspected every 30 min for 1/4-to-2/8-cell division and further dissociated to 1/8-blatomeres. To obtain 2/16-doublets embryos were dissociated at the late 8-cell stage and cultured until 2/16-cell stage.

##### Transplantation of the Apical Domain

Cdx2-EGFP or WT embryos microinjected at the 2-cell stage with Ezrin-mCherry and Myr-palm-IFP mRNAs were used for transplantation. The late 8-cell stage blastomeres were used to obtain the donor cytoplasmic vesicles containing the apical or non-apical domain. For this, polarized blastomeres were deformed into an oval shape by suction into a glass pipette 15-20 μm in diameter attached to a micromanipulator, and sliced with a glass needle to cut off a cytoplasmic vesicle in H-KSOMaa containing 10 μM CCD. As the apical domain is sensitive to CCD treatment, the micromanipulation was performed within 30 min. The cytoplasmic vesicle was then washed in KSOMaa and placed in contact with an as-yet apolar 1/8-cell or an apolar cell of 2/16-doublet. The vesicle fusion was mediated by Sendai virus envelope (Cosmo Bio Co., ISK-CF-001-EX) applied between the blastomere and the vesicle using a glass pipette.

##### Halving the 8-Cell Stage Embryo

To remove blastomeres from the 8-cell stage embryo, a slit was made in the zona pellucida (Tsunoda et al., 1986). Embryos were then transferred into a 10 μl drop of KSOMaa without Ca^2+^ and Mg^2+^ for 15 min at 37°C to loosen cell-cell adhesion. Subsequently 4 blastomeres were sucked out by a glass pipette 15-20 μm in diameter attached to a micromanipulator.

#### Microbeads

PMMA microbeads (Microparticles, PMMA-R-B375) 36 μm in diameter, washed in 0.01% Tween20 (Sigma, P-7949) in DPBS (DPBS-T), were used to establish contact with 8-cell stage blastomeres. To coat microbeads with Cdh1, recombinant mouse Cdh1-Fc chimera protein (RnDsystems, 748-EC-050) was reconstituted at 100 μg/ml in sterile DPBS with Ca^2+^ and Mg^2+^ (Gibco, 14040-091). Protein A-coated PMMA microbeads (Microparticles, PMMA-Protein A-S2976B) 36 μm in diameter, were washed in 0.01% DPBS-T and incubated in 0.8 μg/ml Cdh1 solution at 1600 beads/ml for 90 min at 4°C with 1400 rpm mixing (Thermomixer, Eppendorf). After washing with 0.01% DPBS-T, microbeads were incubated in 1% heat-inactivated BSA (80°C, 10 min; Sigma, A3311) overnight at 4°C to block non-coated sites.

#### Immunofluorescence

Embryos were fixed with 4% paraformaldehyde (PFA; Electron Microscopy Sciences, 19208) in DPBS for 15-30 min at room temperature (RT) and washed with 0.1% DPBS-T. Following permeabilization with 0.25-0.5% TritonX-100 (Sigma, T8787) in DPBS for 30 min at RT, embryos were washed in 0.1% DPBS-T and then blocked in 0.1% DPBS-T for 1 hr at RT or overnight at 4°C. Embryos were then incubated with primary antibodies in the blocking solution overnight at 4°C, washed with 0.1% DPBS-T and incubated with secondary antibodies in 0.1% DPBS-T for 2-3 hrs at RT. After washing with 0.1% DPBS-T, embryos were transferred for microscopy into a 2-10 μl DPBS drop containing DAPI (Molecular Probes, D3571; 1:2000) covered with mineral oil on a glass-bottom dish (MatTek, P356-1.5-20-C). All solutions except for PFA and TritonX-100 were supplemented with 3-5% BSA (Sigma, A9647).

For fixation of the isolated blastomeres the protocol was modified as follows. Isolated blastomeres were fixed in 4% PFA solution supplemented with 0.01% Tween20 (Sigma, P-7949) for 10 min at RT. Washing solution used after fixation was 0.01% DPBS-T supplemented with 0.2% goat serum (Dianova, 005-000-001). Isolated blastomeres were permeabilized with 0.2% TritonX-100 (Sigma, T8787) in DPBS for 10 min at RT. 0.1% DPBS-T supplemented with 5% goat serum was used as the washing solution after permeabilization and as the blocking solution, as we used secondary antibodies raised in goat.

The primary antibodies used in this study were: mouse anti-Cdx2 (BioGenex, MU392-UC; 1:200), goat anti-Sox2 (Santa Cruz Biotechnology, sc-17320; 1:100), rabbit anti-aPKC (Santa Cruz Biotechnology, sc-216; 1:200), mouse anti-aPKC (Santa Cruz Biotechnology, sc-17781; 1:100), rabbit anti-Pard6b (Santa Cruz Biotechnology, sc-67393; 1:200), rat anti-Radixin (1:5000) (Kikuchi et al., 2002), rabbit anti-Amot (1:100) (Hirate et al., 2013), mouse anti-Yap (Abnova, H00010413-M01; 1:100), rat anti-tyrosinated γ-Tubulin (AbD Serotec, MCA77G; 1:200,000), mouse anti-Pcnt (BD, 611814; 1:200) and rabbit anti-SAS4 (1:500) (Basto et al., 2006). The secondary antibodies: donkey anti-goat Alexa Fluor 488 (Life Technologies, A-11055), donkey anti-rat Alexa Fluor 488 (Life Technologies, A-21208), donkey anti-mouse Alexa Fluor 555 (Life Technologies, A31570), donkey anti-rabbit Alexa Fluor 647 (Life Technologies, A31573), goat anti-rabbit Alexa Fluor 488 (Life Technologies, A11008), goat anti-mouse Alexa Fluor 546 (Life Technologies, A21123), goat anti-rat Alexa Fluor 546 (Life Technologies, A11081), goat anti-mouse Alexa Fluor 633 (Life Technologies, A21052) and goat anti-rat Alexa Fluor 633 (Life Technologies, A21094). All secondary antibodies were used at 1:200 dilutions.

#### Live Imaging

Embryos or isolated blastomeres were placed into 2-10 μl KSOMaa drops covered with mineral or silicone oil (Ibidi, 50051) on a glass-bottom dish (MatTek, P356-1.5-20-C) or a plastic-bottom dish (Ibidi, 81151), respectively. For drug treatment experiments embryos or isolated blastomeres were placed in 55 μl of medium in tissue culture treated (Ibidi, 81506) or uncoated dish (Ibidi, 81501), respectively. Time-lapse imaging was performed on LSM780 (Zeiss) using C-Apochromat 40x water objective (Zeiss) at 6-30 min intervals. To compensate for drifting of the embryo during imaging, we used an automatic real-time 3D cell tracking macro, AutofocusScreen (the latest version for ZEN software is available at http://www.ellenberg.embl.de/index.php/software/microscopyautomation) (Rabut and Ellenberg, 2004). Temperature and CO_2_ levels were maintained at 37°C and 5%, respectively, in an incubation chamber specifically designed for the microscope (EMBL). In experiments where non-polarized 1/8-blastomeres expressing Cdx2-EGFP were imaged, the 488 nm laser was not turned on until the end of the 1/8-cell stage to minimize photo-toxicity.

### Quantification and Statistical Analysis

#### Image Analysis

##### Spindle Orientation

Orientation of the mitotic spindle in isolated blastomeres was evaluated by measuring the angle α between the spindle axis, visualized by EGFP-MAP4, and the vector connecting the cell center and the center of the apical domain, visualized by Ezrin-mCherry. The centers of the apical domain and the cell were defined 10 min before NEBD, and the spindle axis at late metaphase/early anaphase. Measurements were made only for those blastomeres that did not substantially move during this time. The cell center was defined by fitting a sphere into the blastomere using Fiji. To identify the apical domain center, coordinates of several points of the apical domain edge were first defined on z-slices in Fiji, into which a circle was fitted (Taubin method, in Matlab). Using the coordinates of the cell center, the center of the fitted circle at the base of the apical domain, and the cell radius, the coordinates of the apical domain center were calculated. Distribution for random spindle orientation is described by the function sin*α* on the interval 0° – 90° (Watanabe et al., 2014).

In the embryo spindle orientation was evaluated by measuring the angle β between the spindle axis, visualized by R26-EGFP-Tuba, and the vector connecting the embryo center and the spindle center. The embryo center was determined by segmenting the embryo based on R26-EGFP-Tuba cytoplasmic signal using IMARIS (Bitplane).

##### Apical Domain

To evaluate the position of the apical domain within the contact-free cell surface of the 8-cell stage embryo, the angle between two unit vectors, **e1** and **e2** (*ω*; Figure S4C) was measured: **e1** is a unit vector connecting the center of the mass of the cell and the center of the contact-free surface, whereas **e2** is another unit vector connecting the center of the mass of the cell and the center of the apical domain.

To compute **e1** and **e2**, we first calculated unit vectors from the center of mass of the cell to voxels on the cell-medium interphase. Subsequently, **e1** and **e2** were calculated as the non-weighted and weighted average of those unit vectors respectively, where the weight was given by fluorescent intensity of Ezrin-mCherry. The Ezrin signal intensity was linearly normalized according to membrane signal to compensate the decay of the signal along the z-axis, followed by subtraction of the minimum signal intensity at the contact-free surface.

A map of the apical domain was generated based on segmented live images. The longitude and latitude (*θ*, *φ*) were assigned to the individual voxel of the segmented surface, with the north-pole specified by the direction from the cell center to the center of the contact-free surface. The normalized Ezrin intensity at (*θ cosφ*, *φ*) was represented as a map (Figure S4A). Change of the apical domain over time (Figure S4B) was shown as a kymograph showing the Ezrin intensity I(*θ*_1_, *φ*) and I(*θ*_1_ + 180°, *φ*) at a longitude *θ*_1_ as a function of *φ* and time. In the vertical axis, the north-pole, *θ* = 90°, is at the center, from which I(*θ*_1_, *φ*) and I(*θ*_1_ + 180°, *φ*) were mapped towards the opposite direction along the axis.

To measure the segregation of the apical domain between two daughter cells, embryos or isolated blastomeres expressing Ezrin-mCherry and fluorescence cell membrane reporter (mG or Myr-palm-IFP, respectively) were imaged during the 8-to-16-cell division. One hour after cytokinesis blastomeres were segmented and a map of the Ezrin intensity normalized to its cytoplasmic background was generated for individual daughters excluding cell-cell interface. Using k-means method, the background level for the Ezrin signal was determined, the sum of intensities of non-background pixels was computed for both daughter blastomeres, and their ratio was calculated.

To evaluate the position of the apical domain in relation to the contact with a microbead, the angle γ between the following two vectors was measured: a vector connecting the cell center and the center of the apical domain, and another connecting the cell center and the microbead center. The center of the apical domain, visualized by Ezrin-mCherry, was determined as described above for the whole embryo 30 min before NEBD. Myr-palm-IFP signal was used to segment the cell membrane and determine the cell center as described above. The center of the bead was defined by fitting a circle into a bead in Fiji. Random distribution of the apical domain position is described as the function *sinα* at 0° – 180°.

##### Cdx2 and Sox2 Expression

To measure the Cdx2 expression level in 2/16-doublets, the mean signal intensity of Cdx2-EGFP and H2B-mCherry were measured in the nucleus of both daughter cells. The measurement was performed on the single middle z-slice through the nucleus 30 min before 2/16-to-4/32-cell division in Fiji. The Cdx2-EGFP signal intensity was normalized to the H2B-mCherry signal.

To measure the Cdx2 and Sox2 expression level in E4.0 WT, *mzPrkci*^*−*/*−*^;*Prkcz*^*−*/*−*^ and *mzCdc42*^−/−^ embryos nuclei of immunofluorescently stained embryos were segmented automatically based on DAPI signal using Fiji, and the mean Cdx2 and Sox2 intensities were measured inside the nuclei. Mitotic cells were excluded from the analysis.

To evaluate the dynamics of Cdx2 expression, nuclei of the Cdx2-EGFP x H2B-mCherry embryo were automatically segmented using Ilastik 1.0 and tracked using StarryNite and AceTree. The mean Cdx2-EGFP intensity was measured inside the nuclei and linearly normalized according to the cytoplasmic signal of interphase R26-EGFP-Tuba embryos imaged under the same condition.

##### Nucleus-to-Cytoplasm Yap Intensity Ratio

The mean Yap intensity was measured in the manually selected area of the nucleus and cytoplasm of immunofluorescently stained 2/16-doublets. The measurement was performed on the slice going thought the nuclei of both daughter cells using Fiji. The nucleus-to-cytoplasm (n/c) Yap intensity ratio of polar cell was divided by n/c Yap intensity ratio of its sister cell.

##### Cell Envelopment

The degree to which one blastomere envelops another was determined by measuring the ratio of the two areas depicted in Figure 1E. Those blastomeres whose interface was nearly parallel to the imaging plane were excluded from the analysis.

##### Image Processing

All displayed images except for Figures 2, 3D, 3E, 5A, 5D, 5F, S1B, S2B, S2C, and S3 were processed with 3D median filter (2x2x2, Fiji). Images in the last time-frame in Figures 1B, 6B, and S5A were enhanced differently from the other time-frames.

#### Statistical Analysis

Graphs were generated and statistical analyses were performed using Matlab and R. No statistical analysis was used to predetermine sample size. Sample sizes, statistical tests and p-values are indicated in the text, figures and figure legends. For statistical analysis data were first analyzed for normality using Shapiro-Wilks test.

## Author Contributions

E.K. and T.H. designed the study. E.K. performed all experiments, except for the characterization of SAS4-EGFP shown in Figure S3, performed by A.C., who also generated SAS4-EGFP BAC transgenic mouse. R.N. developed quantitative image analysis methods, and image analyses were performed by R.N. and E.K. S.F. performed genotyping, including half and single embryo genotyping for mz knockout embryos. N.B. and F.B. generated BAC transgenic ESCs expressing SAS4-EGFP. T.H. and E.K. interpreted the data and wrote the paper with input from all authors.

## Supplemental Information

Document S1. Figures S1–S6 and Table S1

Movie S1. Acquisition of the Apical Domain Predicts the First Lineage Segregation, Related to Figure 1

Movie S2. Isolated 8-Cell Stage Blastomeres Align the Mitotic Spindle to the Apico-Basal Axis, Related to Figure 1

Movie S3. Apical Domain Recruits MTOCs, Related to Figure 3

Movie S4. MTOC Localization in *mzPrkci*^*−*/*−*^;*Prkcz*^*−*/*−*^, and *mzCdc42*^*−*/*−*^ Embryos, Related to Figure 3

Movie S5. Apical Domain Is Sufficient for Initiating Cell-Fate Segregation, Related to Figure 4

Movie S6. Cdh1-Independent Cell Contact Directs Apical Domain Formation, Related to Figure 7

Document S2. Article plus Supplemental Information
