# Supplementary material for: The Apical Domain Is Required and Sufficient for the First Lineage Segregation in the Mouse Embryo
Source: Dev Cell. 2017 Feb 6;40(3):235–247.e7. doi: 10.1016/j.devcel.2017.01.006 (PMC5300053; doi:10.1016/j.devcel.2017.01.006)
Supplement: Document S1. Figures S1–S6 and Table S1 [file mmc1.pdf]

Developmental Cell, Volume *40*

Supplemental Information

The Apical Domain Is Required and Sufficient for the First Lineage Segregation in the Mouse Embryo

Ekaterina Korotkevich, Ritsuya Niwayama, Aurélien Courtois, Stefanie Friese, Nicolas Berger, Frank Buchholz, and Takashi Hiiragi

INVENTORY OF SUPPLEMENTAL INFORMATION

Supplemental Figures

Figure S1. Colocalization of the apical marker and membrane fluorescence signal, related to Figure 1.

Figure S2. Acquisition of the capacity to self-organize the apical domain is temporally controlled, related to Figure 1.

Figure S3. Characterization of the new MTOC-reporter mouse expressing SAS4-EGFP, related to Figure 3.

Figure S4. Apical domain emerges at the center of the contact-free surface, related to Figure 3.

Figure S5. Cell positioning regulates apical domain formation, related to Figure 6.

Figure S6. Cell contact directs formation of the apical domain that in turn induces asymmetric divisions and TE fate specification, related to Figure 7.

Supplemental Tables

Table S1. Genotyping primers, related to STAR Methods.

Supplemental Movies

Movie S1. Acquisition of the apical domain predicts the first lineage segregation, related to Figure 1.

Movie S2. Isolated 8-cell stage blastomeres align the mitotic spindle to the apico-basal axis, related to Figure 1.

Movie S3. Apical domain recruits MTOCs, related to Figure 3.

Movie S4. MTOCs localization in *mzPrkci*$^{-/-}$*;Prkcz*$^{-/-}$ and *mzCdc42*$^{-/-}$ embryos, related to Figure 3.

Movie S5. Apical domain is sufficient for initiating cell fate segregation, related to Figure 4.

Movie S6. Cdh1-independent cell contact directs apical domain formation, related to Figure 7.

SUPPLEMENTAL FIGURES

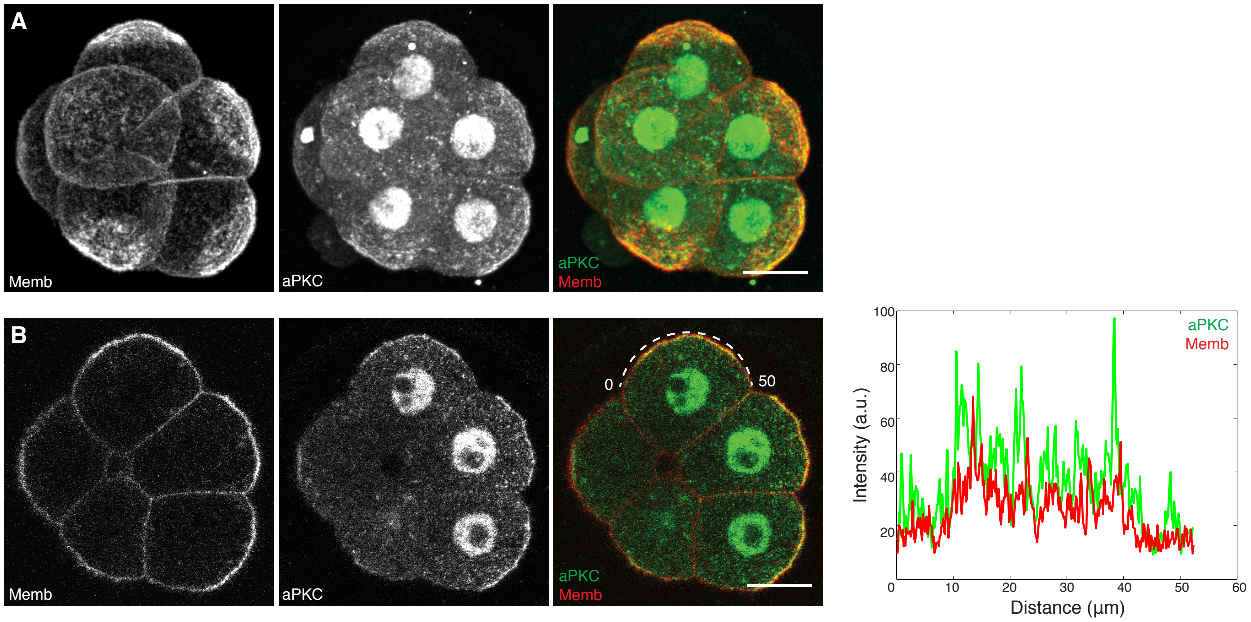

Figure S1. Colocalization of the apical marker and membrane fluorescence signal, related to Figure 1. Maximal intensity projection (MIP; A) and a single-section (B) immunofluorescence image of the 8-cell stage mT embryo fixed and stained for aPKC. Scale bars, 20 μm. Cortical intensity profiles under the dashed line for aPKC and membrane.

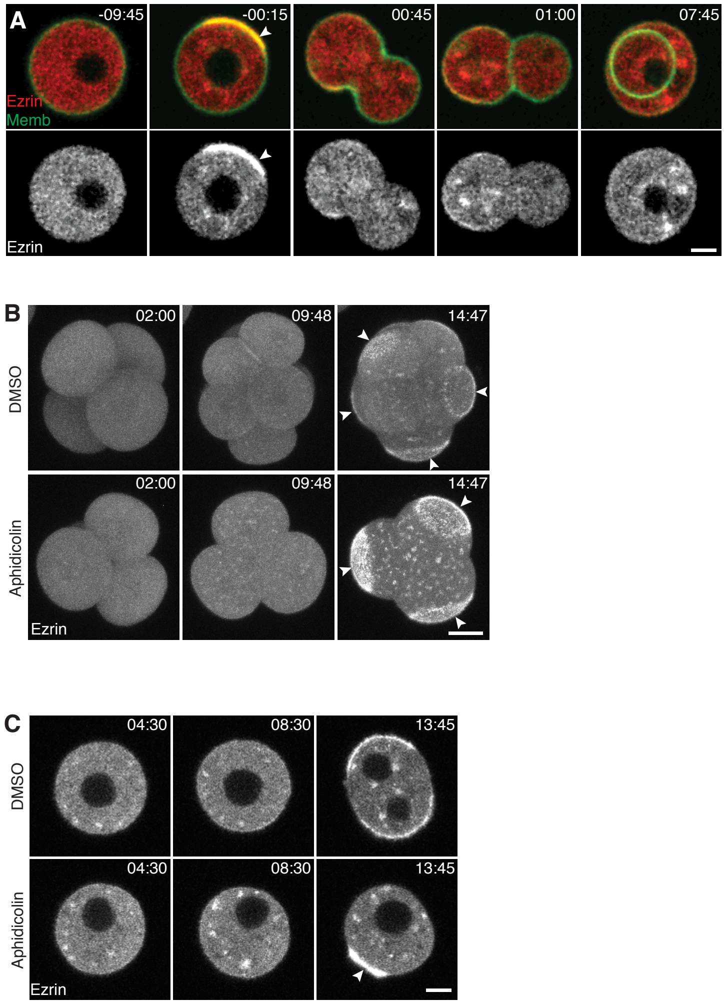

Figure S2. Acquisition of the capacity to self-organize the apical domain is temporally controlled, related to Figure 1.

(A) Cell-autonomous formation of an apical domain in an isolated 8-cell stage blastomere. Time-lapse images of the developing 1/8-cell isolated from an embryo microinjected with Ezrin-mCherry and Myr-palm-IFP (Memb) mRNAs. Time, post-NEBD (hr:min). Scale bar, 10 µm.

(B) Formation of the apical domain is temporally controlled, independent of cell cycle or division. MIP time-lapse images of the 4-cell stage embryo microinjected with Ezrin-mCherry mRNA developing in DMSO (top panel, n = 7 embryos) or 0.5 µg/ml aphidicolin (bottom panel; n = 8 embryos). Time, post-drug treatment (hr:min). Scale bar, 20 µm.

(C) All blastomeres acquire the capacity to form apical domain at the 8-cell stage. Time-lapse images of the 1/8-cell isolated from an embryo microinjected with Ezrin-mCherry mRNA developing in DMSO (top panel; n = 8 of 21 blastomeres did not form an apical domain during the 8-cell stage) or 0.5 µg/ml aphidicolin (bottom panel; n = 13 of 13 blastomeres eventually formed an apical domain during the 8-cell stage). Time, post-drug treatment (hr:min). Scale bar, 10 µm.

Arrowheads, the apical domain.

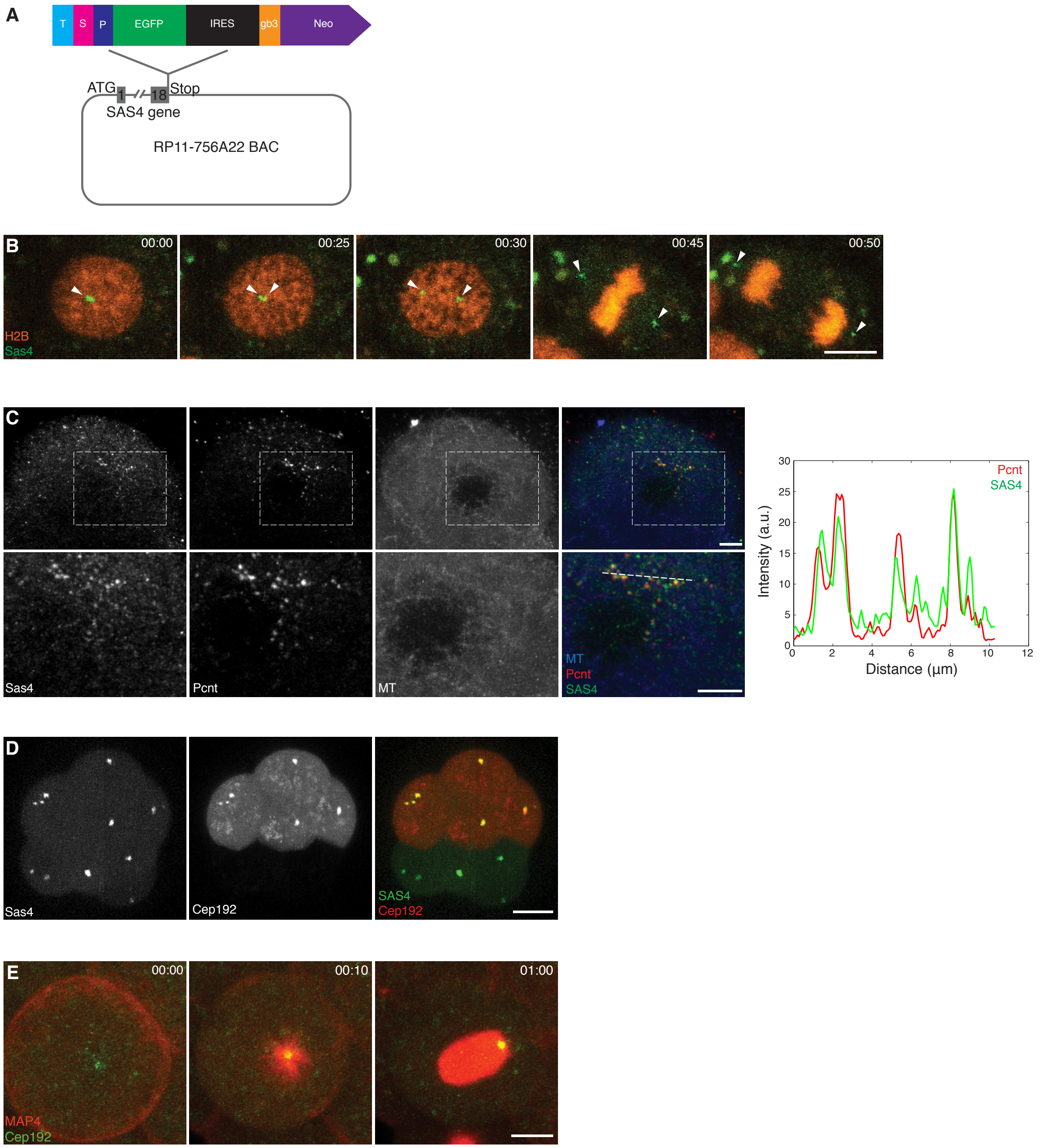

Figure S3. Characterization of the new MTOC-reporter mouse expressing SAS4-EGFP, related to Figure 3.
(A) Design of BAC construct to generate SAS4-EGFP ES cells.
(B) Live image of the E4.0 SAS4-EGFP blastocyst showing SAS4-EGFP signals localized at centrioles (arrowheads). Scale bar, 10 µm.
(C) Colocalization of SAS4 and Pericentrin (Pcnt) nodules at MTOCs shown by immunofluorescence staining of the 8-cell stage embryo and its quantification. Scale bars, 5 µm.
(D) Colocalization of SAS4 and Cep192 in the 8-cell stage SAS4-EGFP embryo microinjected with Cep192-mCherry mRNA in one cell of the 2-cell stage embryo. Scale bar, 20 µm.
(E) Localization of Cep192 at the spindle pole in a cell of the 8-cell stage embryo microinjected with EGFP-MAP4 and Cep192-mCherry mRNAs. Scale bar, 10 µm.
Time (hr:min).

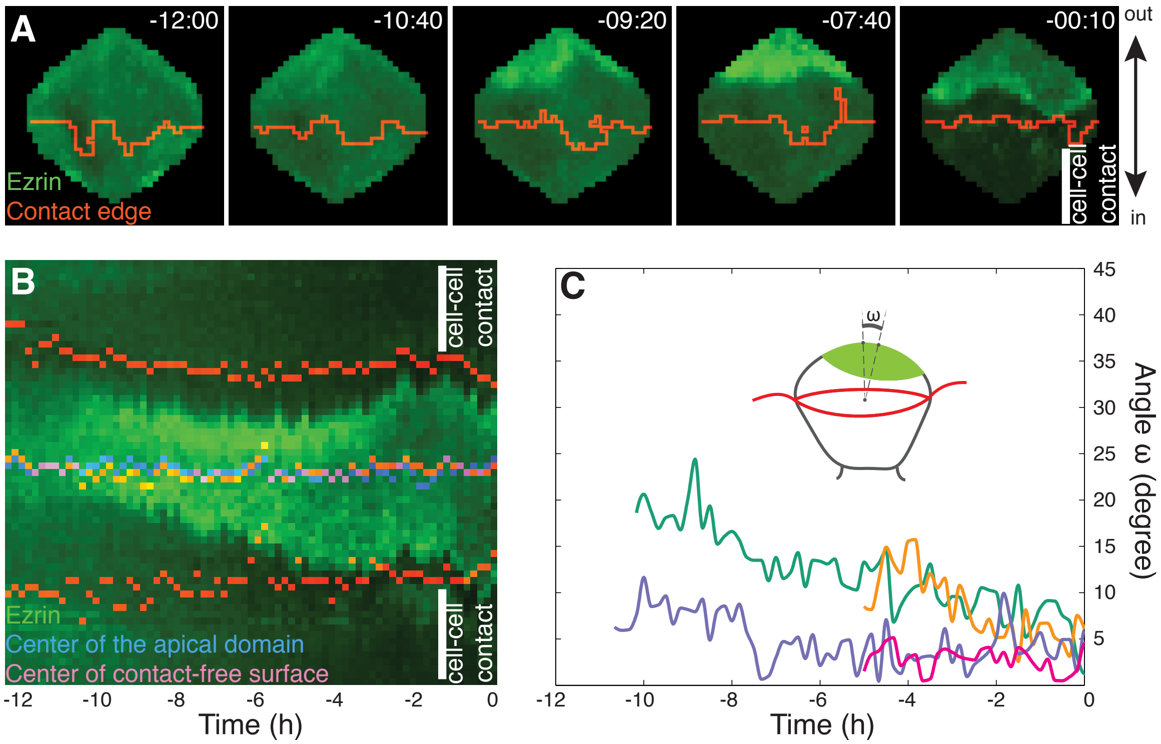

Figure S4. Apical domain emerges at the center of the contact-free surface, related to Figure 3.
(A) Earth-view time-lapse of the apical domain in a cell of 8-cell stage mG embryo microinjected with Ezrin-mCherry mRNA. Red line indicates the edge of cell-cell contact.
(B) Kymograph of the growing apical domain viewed from the north-pole. The center (blue) of the apical domain (green) overlaps with the center (pink) of the contact-free surface (between two red lines).
(C) The deviation angle between the centers of the apical domain and of the contact-free surface decreases as the apical domain grows. Graph based on a representative experiment showing tracks from 4 blastomeres from 2 embryos.
Time, post-NEBD (hr:min).

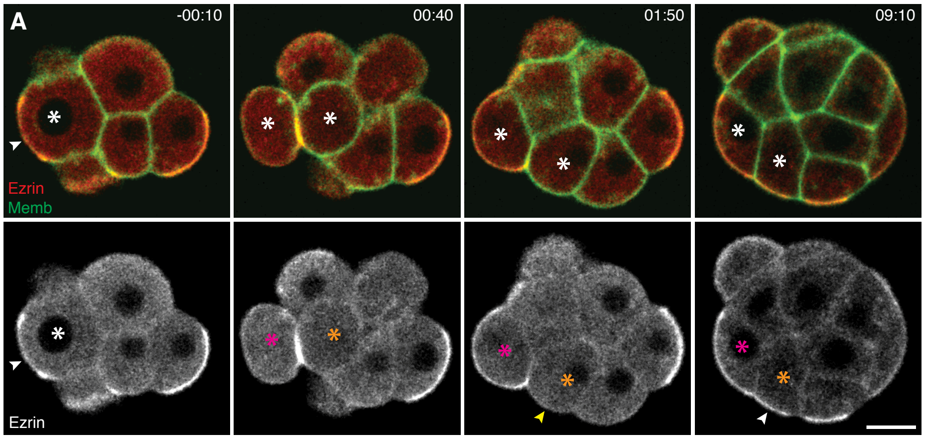

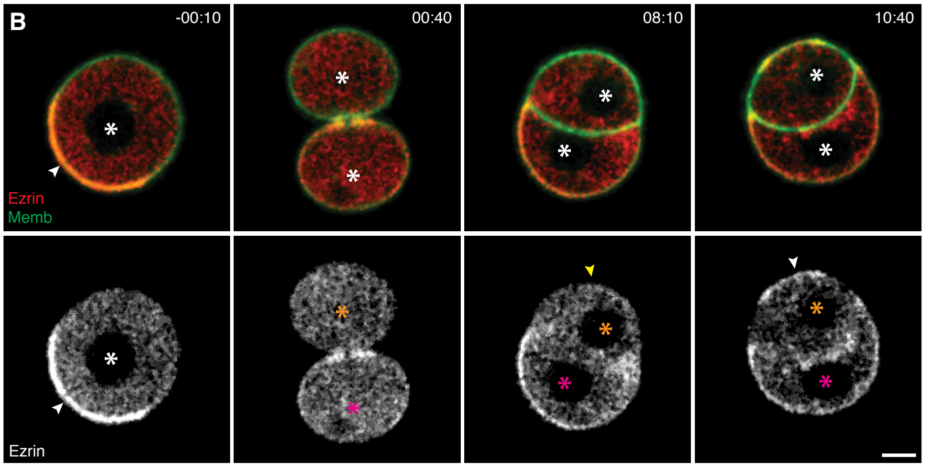

Figure S5. Cell positioning regulates apical domain formation, related to Figure 6.
(A) Time-lapse of asymmetric 8-to-16-cell division (white asterisk; generating cells marked with orange and magenta asterisks) of mG embryo microinjected with Ezrin-mCherry mRNA, in which an apolar cell (orange asterisk and yellow arrowhead) acquires the apical domain (white arrowhead) during the 16-cell stage. The Memb signals are adjusted differently in the last frame (09:10). Scale bar, 20 µm.
(B) Time-lapse of asymmetric division of a 1/8-cell (white asterisk; generating cells marked with orange and magenta asterisks) isolated from an mG embryo microinjected with Ezrin-mCherry mRNA, in which an apolar cell (orange asterisk and yellow arrowhead) acquires the apical domain during the 16-cell stage. Scale bar, 10 µm. Magenta asterisk, polar daughter cell. Time, post-NEBD (hr:min).

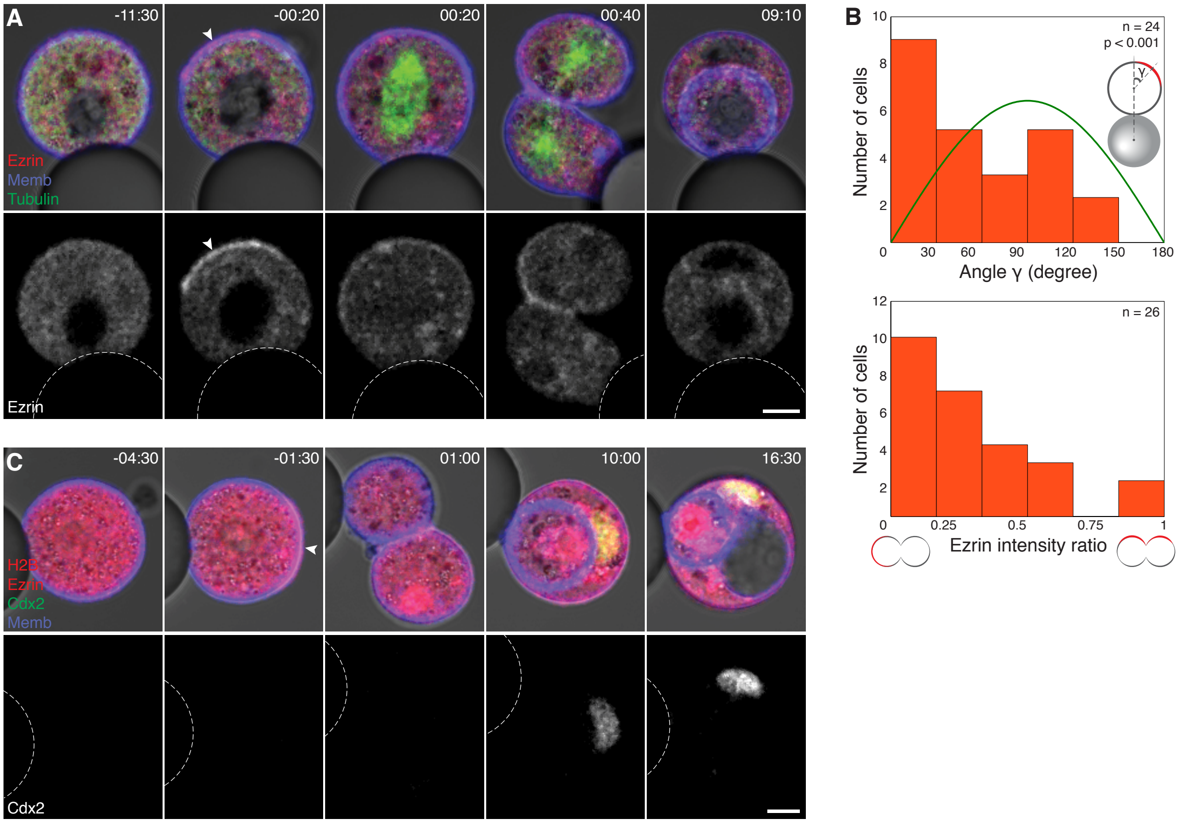

Figure S6. Cell contact directs formation of the apical domain that in turn induces asymmetric divisions and TE fate specification, related to Figure 7.

(A) Time-lapse of the 1/8-cell derived from R26-EGFP-Tuba embryo microinjected with Ezrin-mCherry and Myr-palm-IFP mRNAs developing in contact with a PMMA-bead.

(B) Apical domain forms preferentially within the hemisphere opposite to the cell contact (top; green line, random distribution; Kolmogorov-Smirnov test) and induces its differential distribution to daughters (bottom).

(C) Time-lapse of the 1/8-cell derived from Cdx2-EGFP x R26-H2B-mCherry embryo microinjected with Ezrin-mCherry and Myr-palm-IFP mRNAs developing in contact with a PMMA-bead.

Arrowhead, the apical domain. Dashed line, bead outline. Time, post-NEBD (hr:min). Scale bars, 10 μm.

SUPPLEMENTAL TABLES

Table S1. Genotyping primers, related to STAR Methods.

Mouse line	Primer name	Sequence	PCR product size, bp
Cdx2-EGFP knock-in	Cdx2-Fw	ATGGTTCCGTTCCCTGGTTC	WT allele ~1400 Knock-in allele ~750
	GFP-R	GCGGACTTGAAGAAGTCGTGCTGCTT	
	Cdx2-Ex3	AGGCTTGTTTGGCTCGTTACA C	
R26-EGFP-Tuba and R26-H2B-mCherry	R26-P3	TCCCTCGTGATCTGCAACTCCAGTC	WT allele = 217 Knock-in allele = 270
	R26-P4	AACCCCAGATGACTACCTATCCTCC	
	R26-P6	GCTGCAGGTCGAGGGACC	
mTmG	oIMR7318	CTCTGCTGCCTCCTGGCTTCT	WT allele = 330 Knock-in allele = 250
	oIMR7319	CGAGGCGGATCACAAGCAATA	
	oIMR7320	TCAATGGGCGGGGGTCGTT	
SAS4-EGFP BAC	SAS4_F	CTGCTAAATTCGAACGCCAGC	828
	SAS4_R	CGTCCATGCCGAGAGTGATC	
$Prkci^{tm1.1Kido}$	APKCNF	CTAGAACTGAACCCAGGCCCTCTG	WT allele = 249 Floxed allele = 300 Deletion ~ 170
	APKCFF	CATGCAGTGTGCTGGCATAGCCACC	
	APKCR	CAAAGCCCTGCTCTCCTAGAGCCTG	
$Prkcz^{tm1.1Cda}$	Prkcz-1	AAAGGGGCACTGGAGATTAAACCC	WT allele = 272 Deletion = 322
	Prkcz-2	GGAATTACCACACGACCTAGCAGC	
	Prkcz-5	GCCGTGTGAAATTGTGCTTCAGTG	
$Cdc42^{tm1Brak}$	Cdc42 del	TGAAACACTTGAGGCCATGA	WT allele = 994, Floxed allele ~1200 Deletion ~680
	MR 6	TCTGCCATCTACACATACAC	
$Cdh1^{tm2Kem}$	pE10.2	CTTATACCGCTCGAGAGCCGG A	WT allele= 900 Floxed allele = 980
	pE11as.2	GTGTCCCTCCAAATCCGATA	
	pI5s1	GAATTCTGAACATCATTATCAGTATTTA	Deletion ~350
	pI10as.3	TGACACATGCCTTTACTTTAGT	
ZP3-Cre	Cre_upper	TGCTGTTTCACTGGTTGTGCGGCG	~250
	Cre_lower	TGCCTTCTCTACACCTGCGGTGCT	

SUPPLEMENTAL MOVIES

Movie S1. Acquisition of the apical domain predicts the first lineage segregation, related to Figure 1.

Time-lapse images of the developing 1/8-cell derived from Cdx2-EGFP (green) x R26-H2B-mCherry (red) embryo microinjected with Myr-palm-IFP (grey) mRNA. Time, post-NEBD (hr:min). Scale bar, 10 µm.

Movie S2. Isolated 8-cell stage blastomeres align the mitotic spindle to the apico-basal axis, related to Figure 1.

Time-lapse images of 1/8-cell isolated from R26-EGFP-Tuba (green) embryo microinjected with Ezrin-mCherry (red) and Myr-palm-IFP (blue) mRNAs. Time, post-NEBD (hr:min). Scale bar, 10 µm.

Movie S3. Apical domain recruits MTOCs, related to Figure 3.

Time-lapse images of the 8-cell stage SAS4-EGFP (green) transgenic embryo microinjected with Ezrin-mCherry (red) mRNA. Time, 00:00 is 68 hrs post-hCG (hr:min). Scale bar, 20 µm.

Movie S4. MTOCs localization in *mzPrkci*$^{-/-}$*;Prkcz*$^{-/-}$ and *mzCdc42*$^{-/-}$ embryos, related to Figure 3.

Time-lapse images of *mzPrkci*$^{-/-}$*;Prkcz*$^{-/-}$ x SAS4-EGFP (green) x mT (red; left panel) and *mzCdc42*$^{-/-}$ x SAS4-EGFP x mT (right panel) embryos developing from the 8- to 16-cell stage. Time, post-NEBD (hr:min). Scale bars, 20 µm.

Movie S5. Apical domain is sufficient for initiating cell fate segregation, related to Figure 4.

(1) Time-lapse images of an 8-cell stage blastomere isolated from the embryo microinjected with Ezrin-mCherry (red) and Myr-palm-IFP (blue) mRNAs. An apolar blastomere fused with a cell fragment containing the apical domain integrated and maintained this apical domain, as monitored by the Ezrin signal.

(2) Time-lapse images of an 8-cell stage blastomere isolated from Cdx2-EGFP (green) embryo microinjected with Ezrin-mCherry (red) and Myr-palm-IFP (blue) mRNAs, developing after integration of a cell fragment derived from an 8-cell stage blastomere containing the apical domain.

Time, post-NEBD (hr:min). Scale bar, 10 µm.

Movie S6. Cdh1-independent cell contact directs apical domain formation, related to Figure 7.

Z-scanning sections of the live 8-cell stage *mzCdh1*$^{-/-}$ embryo microinjected with Ezrin-mCherry and Myr-palm-IFP mRNAs. Z position (µm). Scale bar, 20 µm.
