# Supplementary figures and images for: The Apical Domain Is Required and Sufficient for the First Lineage Segregation in the Mouse Embryo
Source: Dev Cell. 2017 Feb 6;40(3):235–247.e7. doi: 10.1016/j.devcel.2017.01.006 (PMC5300053; doi:10.1016/j.devcel.2017.01.006)

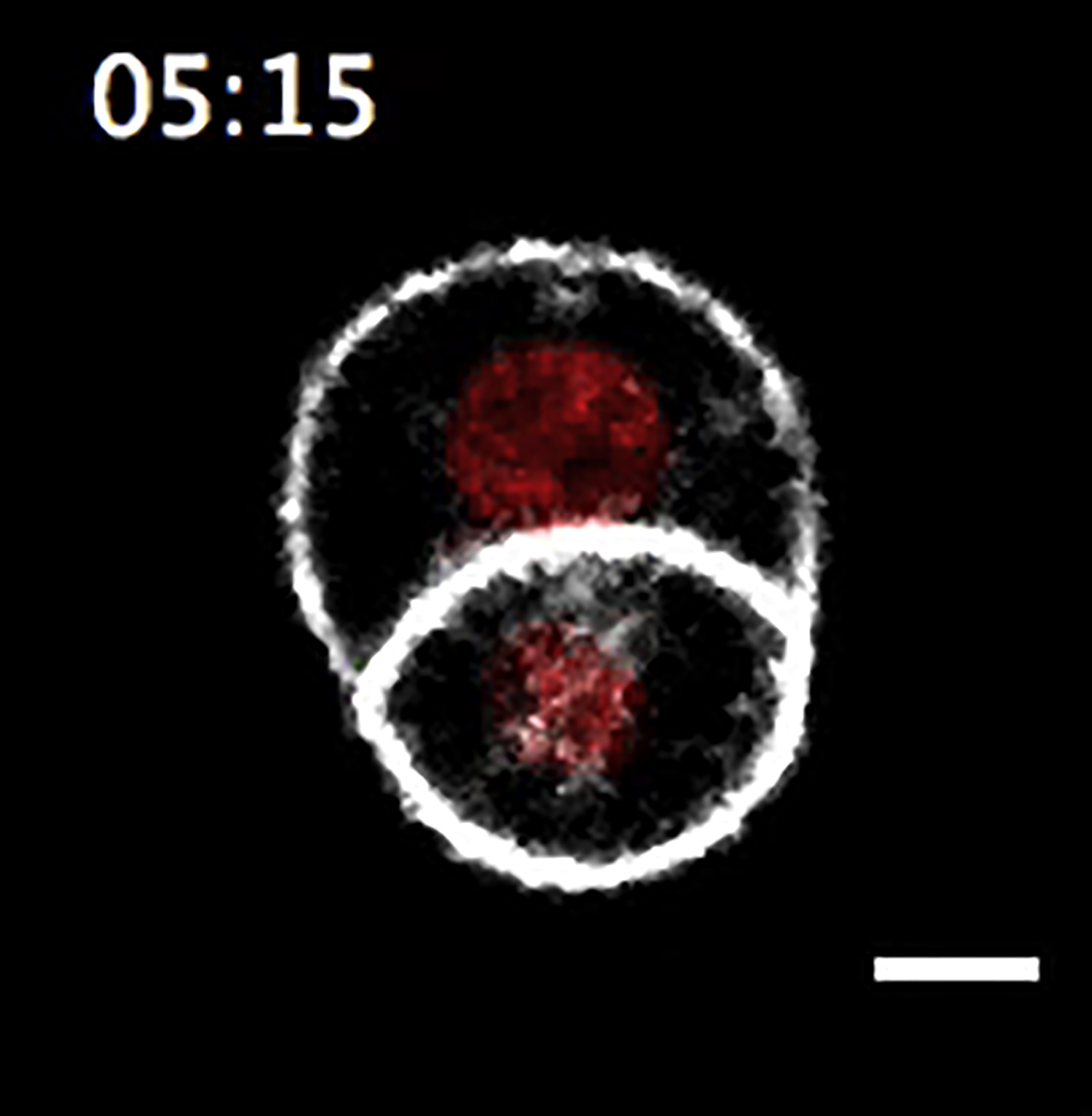

Supplement: Movie S1. Acquisition of the Apical Domain Predicts the First Lineage Segregation, Related to Figure 1 [file mmc2.jpg]

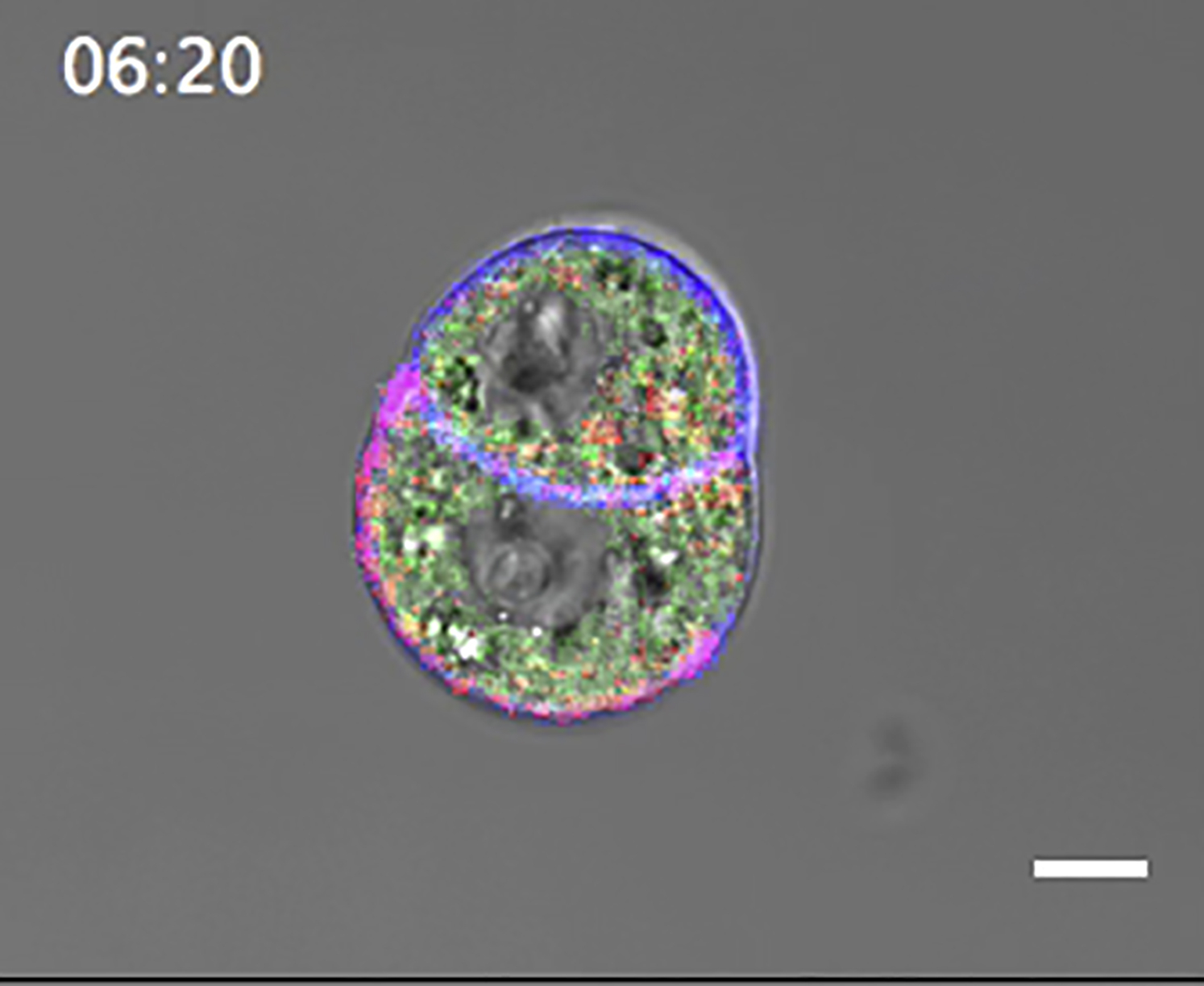

Supplement: Movie S2. Isolated 8-Cell Stage Blastomeres Align the Mitotic Spindle to the Apico-Basal Axis, Related to Figure 1 [file mmc3.jpg]

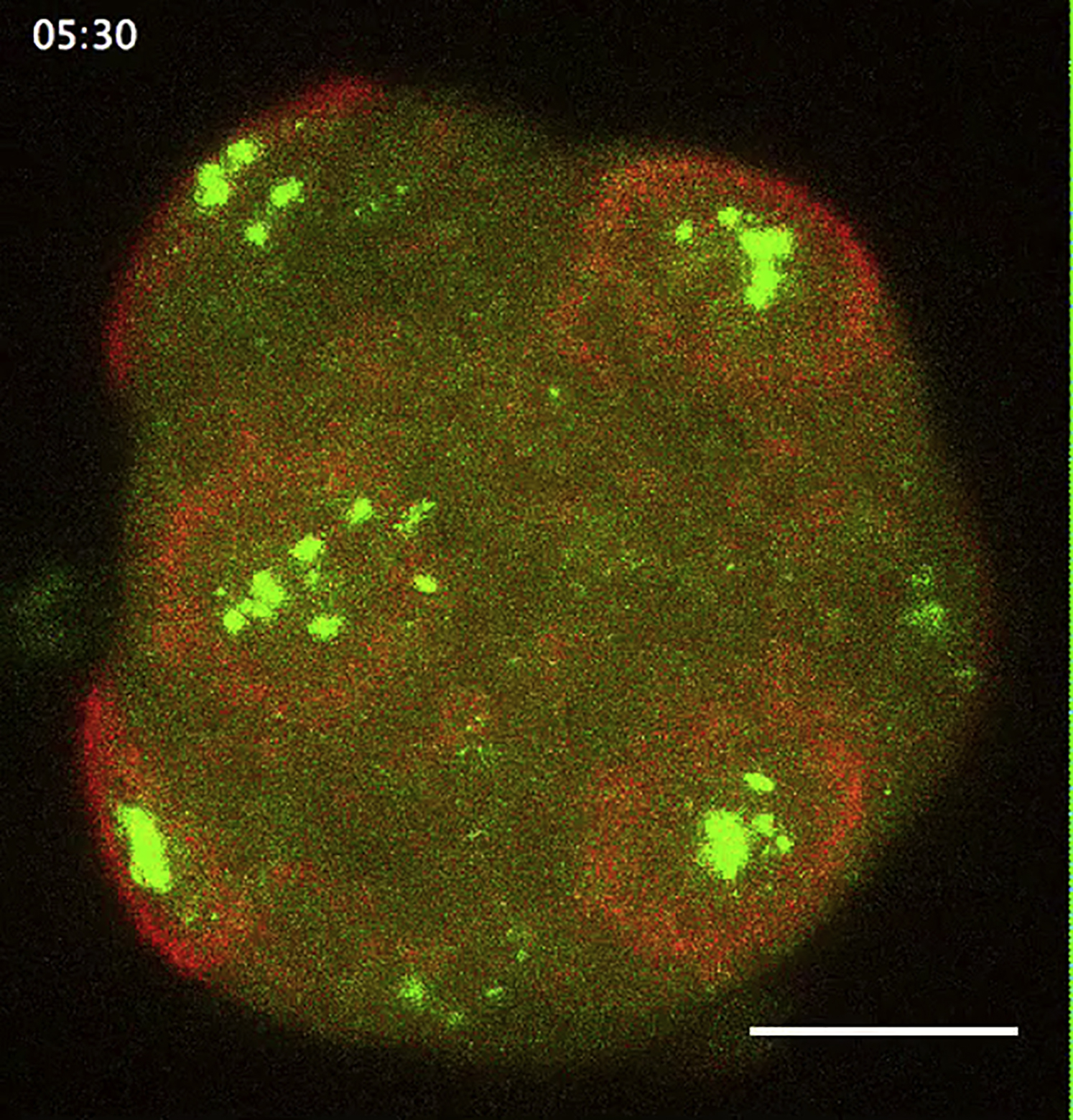

Supplement: Movie S3. Apical Domain Recruits MTOCs, Related to Figure 3 [file mmc4.jpg]

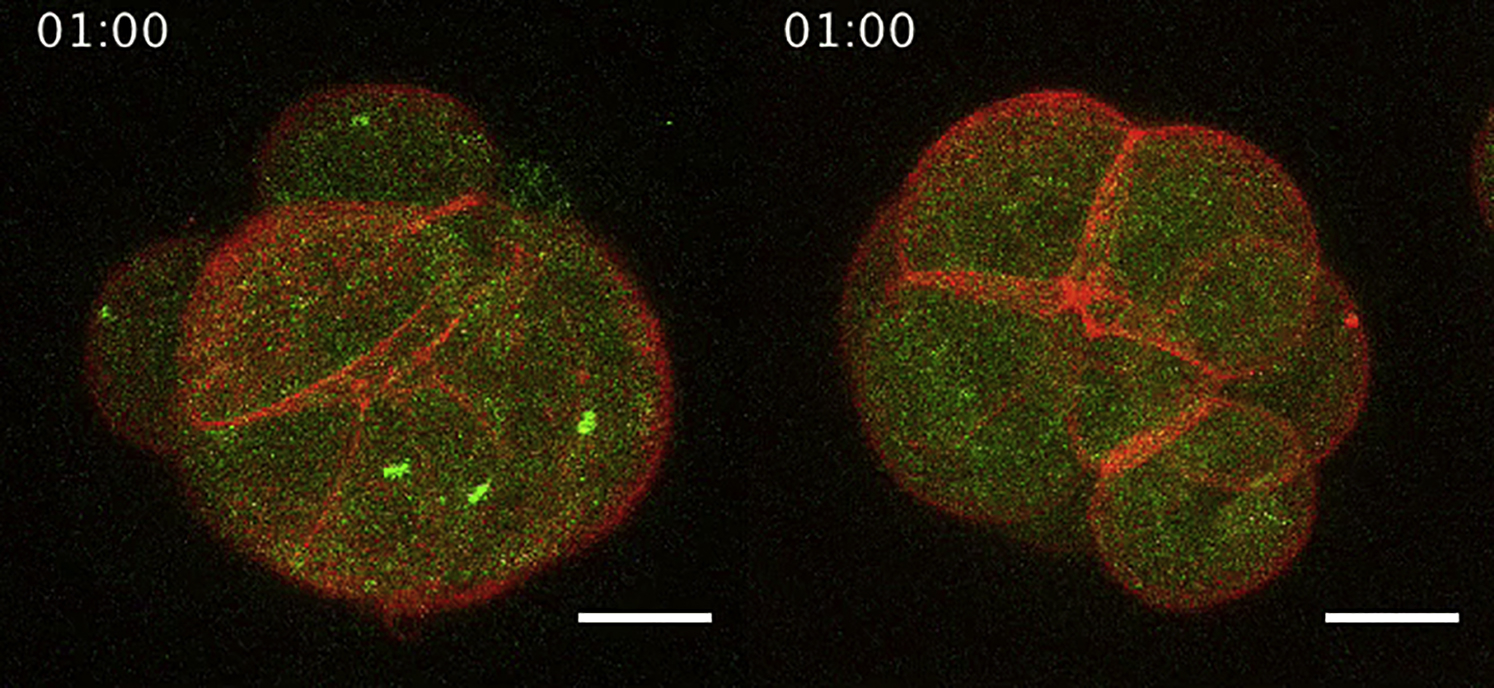

Supplement: Movie S4. MTOC Localization in mzPrkci−/−;Prkcz−/−, and mzCdc42−/− Embryos, Related to Figure 3 [file mmc5.jpg]

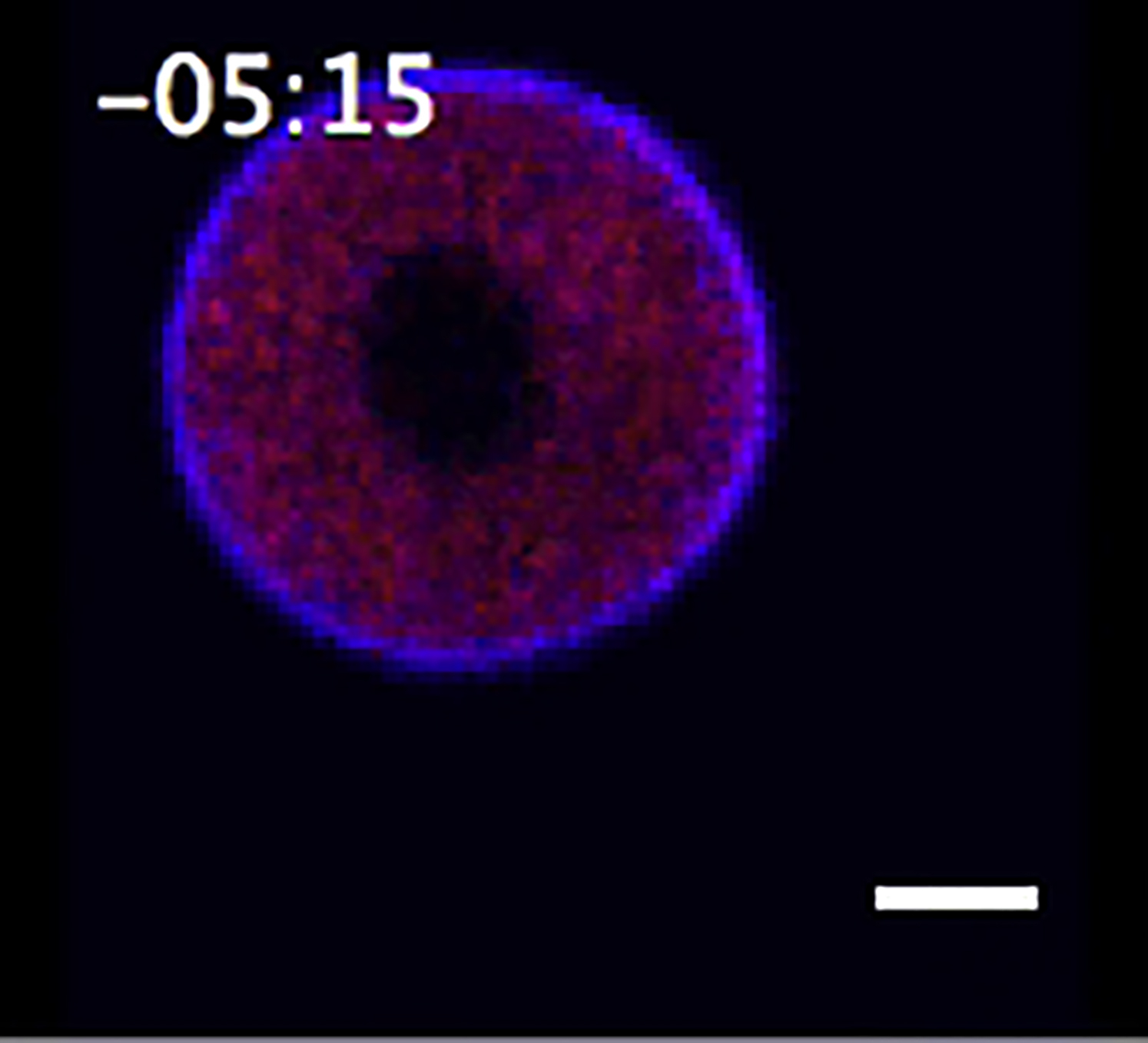

Supplement: Movie S5. Apical Domain Is Sufficient for Initiating Cell-Fate Segregation, Related to Figure 4 [file mmc6.jpg]

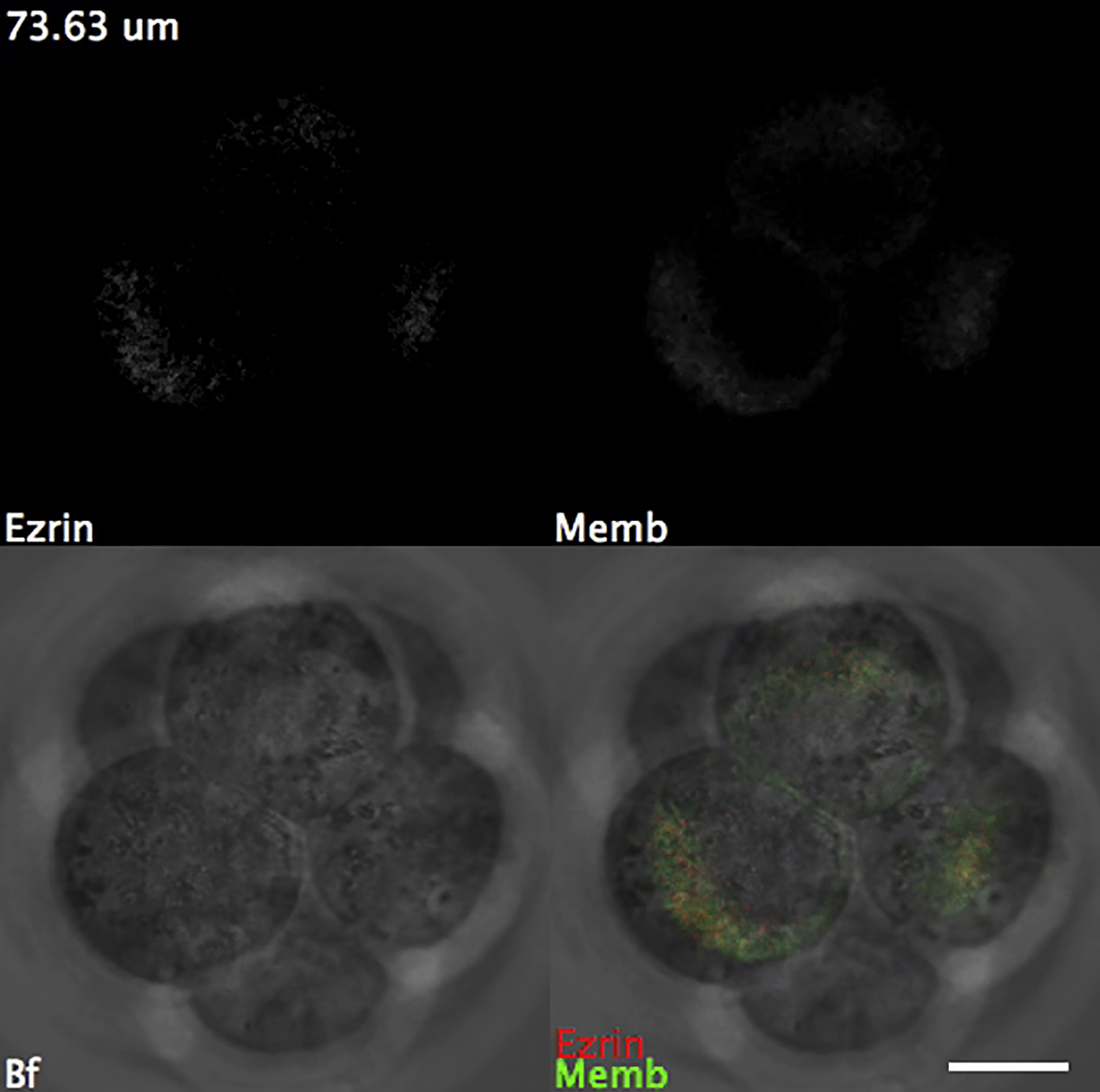

Supplement: Movie S6. Cdh1-Independent Cell Contact Directs Apical Domain Formation, Related to Figure 7 [file mmc7.jpg]
